# Predictive Role of NEK6 in Prognosis and Immune Infiltration in Head and Neck Squamous Cell Carcinoma

**DOI:** 10.3389/fendo.2022.943686

**Published:** 2022-07-11

**Authors:** Zhi-Min Yang, Bing Liao, Si-Si Yang, Tong Su, Jing Zhang, Wei-Ming Wang

**Affiliations:** ^1^ Department of Oral and Maxillofacial Surgery, Center of Stomatology, Xiangya Hospital, Central South University, Changsha, China; ^2^ Institute of Oral Precancerous Lesions, Central South University, Changsha, China; ^3^ Research Center of Oral and Maxillofacial Tumor, Xiangya Hospital, Central South University, Changsha, China

**Keywords:** NEK6, prognosis, immune infiltration, HNSCC, immune checkpoints

## Abstract

Head and neck squamous cell carcinoma (HNSCC), as one of the common malignant tumors, seriously threatens human health. NEK6 (Never in Mitosis A (NIMA) related kinases 6), as a cyclin, promotes cancer cell proliferation and cancer progression. However, the prognostic value of NEK6 and its correlation with immune cell infiltration in HNSCC remain unclear. In this study, we comprehensively elucidated the prognostic role and potential function of NEK6 expression in HNSCC. The expression of NEK6 was significantly up-regulated by immunohistochemistry in HNSCC. Upregulation of NEK6 expression in gene expression studies predicts poor prognosis in HNSCC patients. The results of Gene Ontology (GO), Kyoto Encyclopedia of Genes and Genomes (KEGG) and Gene set variation analysis indicated that NEK6 is mainly involved in extracellular matrix metabolism and EMT processes. The expression of NEK6 increased with the level of immune cell infiltration and the expression of various immune checkpoints. In conclusion, NEK6 may serve as a candidate prognostic predictor and may predict the response of HNSCC patients to immunotherapy.

## Introduction

Head and neck squamous cell carcinoma (HNSCC), as one of the common malignant tumors, seriously threatens human health (1). HNSCC metastasizes to lymph nodes *via* lymphatic channels at an early stage. Due to the lack of active treatment opportunities or the high recurrence and metastasis rate after treatment, the 5-year survival rate of advanced patients is less than 30% (2). In recent years, with the clinical application of new drugs (cetuximab and immune checkpoint inhibitors), the survival of some HNSCC patients has been prolonged (3, 4). However, more than 70% of patients with advanced HNSCC still do not benefit from these drugs (3). Therefore, it is necessary to develop new valuable biomarkers to predict the therapeutic effect of HNSCC.

Previous studies have shown that cell cycle-related proteins promote tumor cell proliferation by regulating cell mitosis and promote invasion and therapy resistance (5). NEK6 (Never In Mitosis A (NIMA) related kinases 6), a cyclin, promotes the invasion and metastasis of various cancers by regulating cell proliferation and apoptosis (6). NEK6 is highly expressed in colorectal cancer, breast cancer, gastric cancer, prostate cancer, liver cancer, ovarian cancer, and thyroid cancer (6–11).

Since the first immune checkpoint inhibitor (ICI) drug, ipilimumab, was approved for clinical use by the US FDA in March 2011, ICIs (PD-1, PD-L1, and CTLA4) have shown promising therapeutic effects in a variety of tumors, suggesting that Inhibition of tumor-specific immunity prevents the occurrence and development of tumors (3, 12). However, only a minority of cancer patients have complete and durable responses to ICIs therapy, and most patients still do not benefit from ICIs therapy (13). Therefore, improving the response rate of immunotherapy in immunotherapy research is one of the research hotspots. Whether NEK6 expression in HNSCC is associated with immune cell infiltration has been poorly studied.

Therefore, in this study, a variety of bioinformatics methods and HNSCC tissue specimens were used to comprehensively measure the relationship between NEK6 expression and prognosis in HNSCC. The relationship between tumor immune cell infiltration and immune checkpoint molecule expression and NEK6 expression was also further analyzed. These results provide new insights into the function of NEK6 and new targets for the diagnosis and prognosis of HNSCC.

## Methods

### Data Collection and Processing

Extracted pan-cancer sequencing data from The Cancer Genome Atlas (TCGA) and Broad Institute Cancer Cell Line Encyclopedia (CCLE) for analysis through their portals. Use the rma function in the R package (R studio version: 1.2.1335, R version: 3.6.1) (http://www.r-project.org/https://www.rstudio.com/) for the whole data set to filter. Missing and duplicated results were removed and transformed by log2(TPM +1). Patients’ age, gender, tumor stage, and clinical stage were retrieved from the portal, along with other clinical data.

### Survival Analysis

In the R setting, Cox regression analysis was used to analyze the relationship between NEK6 expression and survival in HNSCC patients. After the patients were divided into NEK6 high-expression group and low-expression group by the optimal separation method, the Kaplan-Meier method was used to create the survival curve of HNSCC patients. Survival was studied using the Survival ROC and Survival in the R package (rdocumentation.org/packages/survival). Differences between curves were examined using the log-rank test, P values less than 0.05 were considered significant.

### Immune Cell Infiltration Enrichment and Correlation Analysis of Immune Checkpoints

Tumor Immune Estimation Resource (TIMER) is a database-driven web application that calculates immune cell infiltration fractions for six major immune cell types, including B cells, CD4+, T cells, CD 8+, T cells, macrophages, Neutrophils and dendritic cells. Retrieve and examine infiltration data to see if there is a link between NEK6 expression and infiltration. Similarly, TIMER can also retrieve and examine whether there is a link between NEK6 expression and immune checkpoint gene expression.

### NEK6-Related Gene Enrichment Analysis

We searched the STRING database (https://string-db.org/) using individual protein names (“NEK6”) and organisms (“Homo sapiens”). After that, we set the following main parameters: the minimum interaction score required [“Low confidence (0.150)”], the meaning of the network edge (“evidence”), the maximum number of interactors to display (“No more than 50 interactors” “ in the first shell) and an active interaction source (“experimental”). Finally, we searched for NEK6-binding proteins that had been determined experimentally. In addition, two sets of data (Kyoto Encyclopedia of Genes and Genomes) were integrated for KEGG pathway analysis. We collected data for functional annotation graphs by uploading gene lists to DAVID, a database for annotation, visualization, and integrated discovery, using parameters for the selected identifier (“OFFICIAL_GENE_SYMBOL”) and species (“Homo sapiens”). Finally, enriched pathways are displayed using the R packages “tidyr” and “ggplot2”. Additionally, we use the R package “clusterProfiler” to run GO.

### Gene Set Variation Analysis

Gene set variation analysis (GSVA) was based on the MsigDB database (https://www.gsea-msigdb.org/gsea/msigdb/index.jsp) HALLMARK pathway dataset.

### Immunohistochemistry

The present study was approved by the Medical Ethics Committee of Xiangya Hospital, Central South University (Hunan, China) and was performed according to the Declaration of Helsinki guidelines on experimentation involving human subjects. Written informed consent was obtained from all participants. The sections of HNSCC were deparaffinized in xylene and rehydrated in a graded series of ethanol and double-distilled water before subjected to heat-induced antigen retrieval. After incubated with primary antibodies (NEK6 1:200 abcam, USA ab117986) overnight at 4°C, the secondary antibody was incubated at room temperature for 1 hr. Image-Pro Plus 6.0 (Media Cybernetics, Inc.) was used to calculate the density determination.

### Statistical Analysis

Spearman’s correlation test was used to analyze the association between NEK6 expression and targets. According to whether the samples were paired or not, the comparison between normal tissue and cancer tissue was performed by two-group t-test. All graphics were created using R software. Data are reported as mean ± SD. Differences were defined as statistically significant if P-value < 0.05.

## Results

### Increased NEK6 Expression in HNSCC

**Figure 1:**
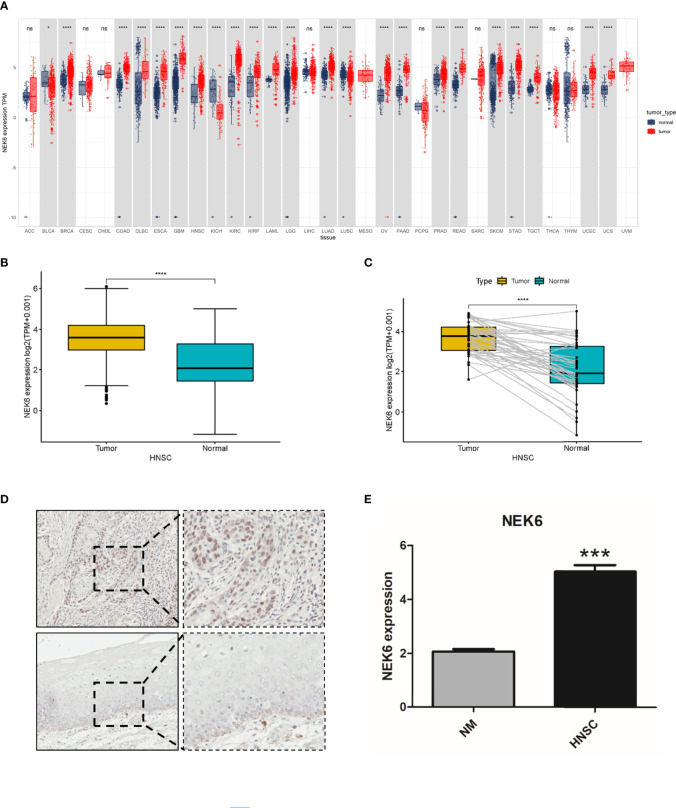
Expression of NEK6 in HNSCC. **(A)** NEK6 expression in different types of cancer was investigated with the TIMER database. **(B)** Analysis of NEK6 expression in HNSCC and adjacent normal tissues in the TCGA database. **(C)** TCGA database and statistical analyses of HNSCC expression in HNSCC tissues and paired adjacent normal tissues. **(C)** Immunohistochemical staining of NEK6 was performed in HNSCC and normal mucosa. Representative images are shown. Scare bars, 50 mM. **(D, E)** The staining was quantified, as shown. ***p < 0.001.

Compared with corresponding normal tissues (**Figure 1A**), the expression of NEK6 are increased in breast invasive carcinoma (BRCA), colon adenocarcinoma (COAD), Lymphoid Neoplasm Diffuse Large B-cell Lymphoma, esophageal carcinoma (ESCA), Glioblastoma multiforme(GBM), HNSCC, kidney chromophobe (KICH), and Kidney renal papillary cell carcinoma (KIRP). However, NEK6 expression are decreased in Bladder Urothelial Carcinoma (BLCA), renal chromophobe cells (KICH), and Lung squamous cell carcinoma(LUSC). Analysis of NEK6 expression in HNSCC samples and adjacent normal tissue using data gained directly from The Cancer Genome Atlas (TCGA). The results showed that the expression of NEK6 was significantly increased in HNSCC tissues compared to normal tissues (**Figure 1B**). Furthermore, the expression of NEK6 was significantly increased in HNSCC compared with paired normal samples (**Figure 1C**). Immunohistochemically results also showed similar results; the expression of NEK6 was significantly increased in HNSCC. (**Figures 1D, E**).

### NEK6 Expression Was Associated With Prognosis in Patients With HNSCC

**Figure 2:**
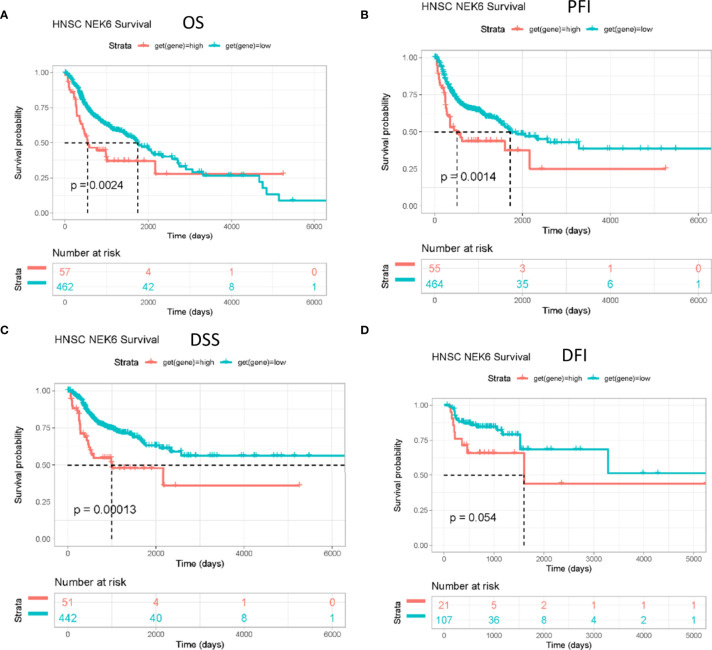
Prognostic value of NEK6 in HNSCC. Survival curves for **(A)** OS, **(B)** PFI, **(C)** DSS, **(D)** DFI were displayed using a Kaplan-Meier plotter.

OS (overall survival), PFI (progression-free interval) and DSS (disease-related survival) of patients with high NEK6 gene expression in HNSCC were worse than those with low expression (**Figures 2A** **–C**) according to the Kaplan-Meier plotter database. However, NEK6 expression was not correlated with DFI (disease free interval) (**Figure 2D**).

### Analysis of NEK6 and Its Co-Expressed Genes in HNSCC

**Figure 3:**
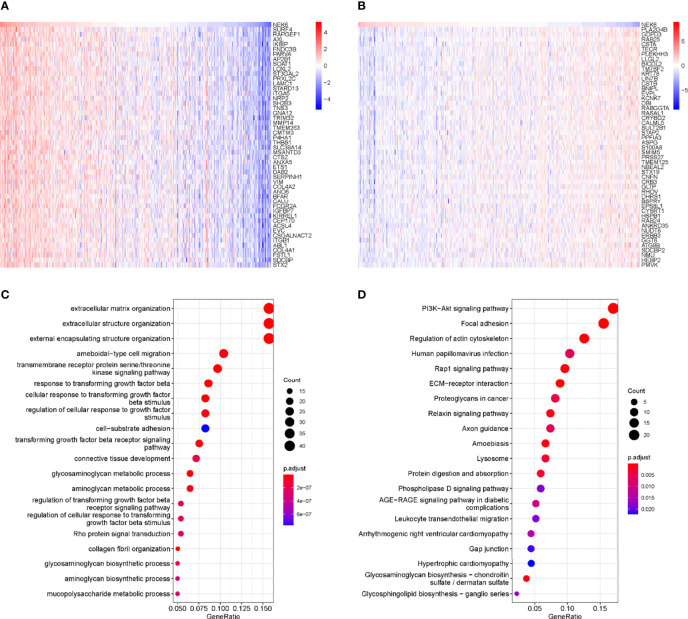
GO and KEGG enrichment analysis of NEK6. **(A)** Top 50 genes negatively correlated with NEK expression levels in HNSCC. **(B)** Top 50 genes positively and negatively correlated with NEK expression levels in HNSCC. **(C)** Top 20 BP enrichment items in HNSCC. **(D)** Top 20 KEGG-enriched pathways in HNSCC.

**Figures 3A, B** show the top 50 genes positively and negatively associated with NEK6 in HNSCC. KEGG and GO enrichment analysis was performed using 300 genes positively correlated with NEK6 to explore NEK6-related pathways and biological functions. In terms of BP, NEK6 was enriched in functions such as extracellular matrix organization, extracellular structure organization, external encapsulating structure organization, ameboidal-type cell migration (**Figure 3C**). The top 20 KEGG pathways of NEK6 and its related genes are shown in **Figure 3D**. The most significant correlation is the PI3K-Akt signaling pathway among these pathways. In addition, pathways such as focal adhesion and regulation of actin cytoskeleton were also significantly positively correlated with NEK6 expression.

### Analysis of NEK6-Related Signaling Pathways by GSVA

**Figure 4:**
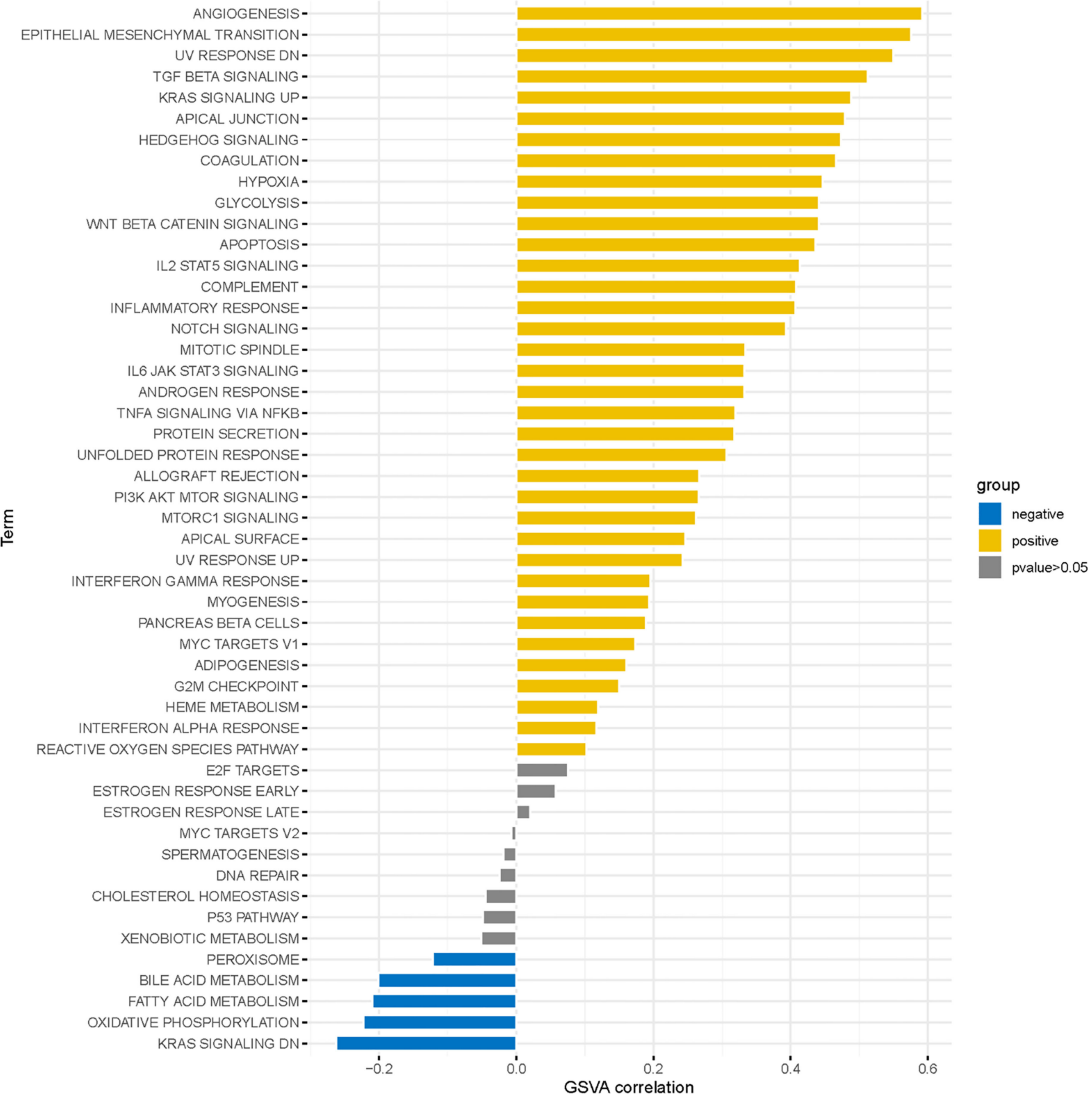
GSVA enrichment analysis for NEK6.

The top 20 signaling pathways affected by NEK6 were mainly enriched in angiogenesis, epithelial mesenchymal transition, and TGFβ pathways (**Figure 4**). These results strongly implicate NEK6 in the regulation of HNSCC cell migration and motility

### Correlation Analysis of NEK6 Expression and Infiltrating Immune Cells

**Figure 5:**
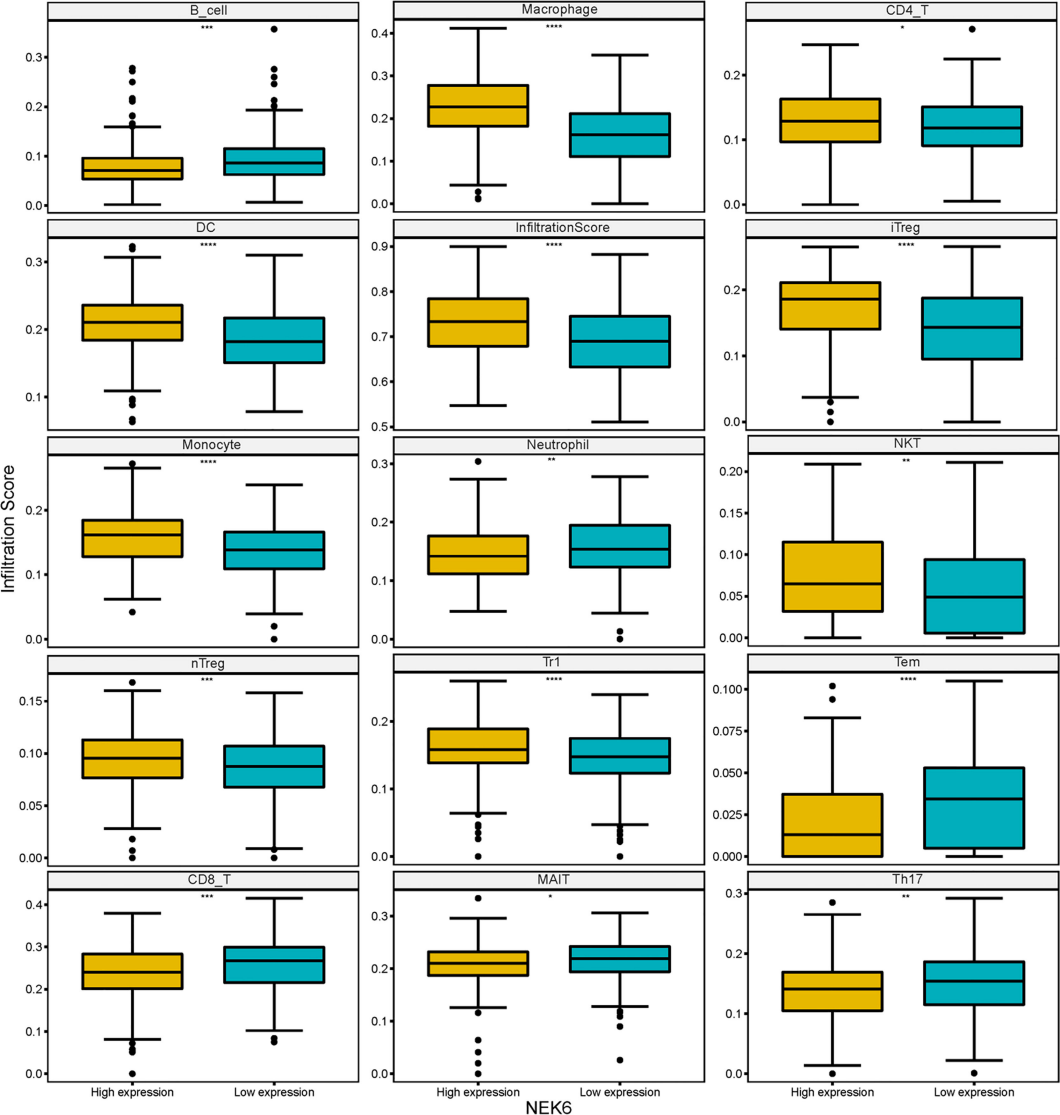
Immune infiltrating cells associated with NEK expression in HNSCC.

NEK6 positively correlated with infiltrating levels of B cells, CD8+ T cells, neutrophil cells, mucosa-associated invariant T cells (MAIT), TEM cells, and Th17 cells, and negatively correlated with DCs, monocyte cells, nTreg cells, Macrophage, Tr1, CD4 T cells, iTreg cells, and NKT infiltration (**Figure 5**).

### Correlation of NEK6 Expression With Expression of Various Immune Checkpoints

**Figure 6:**
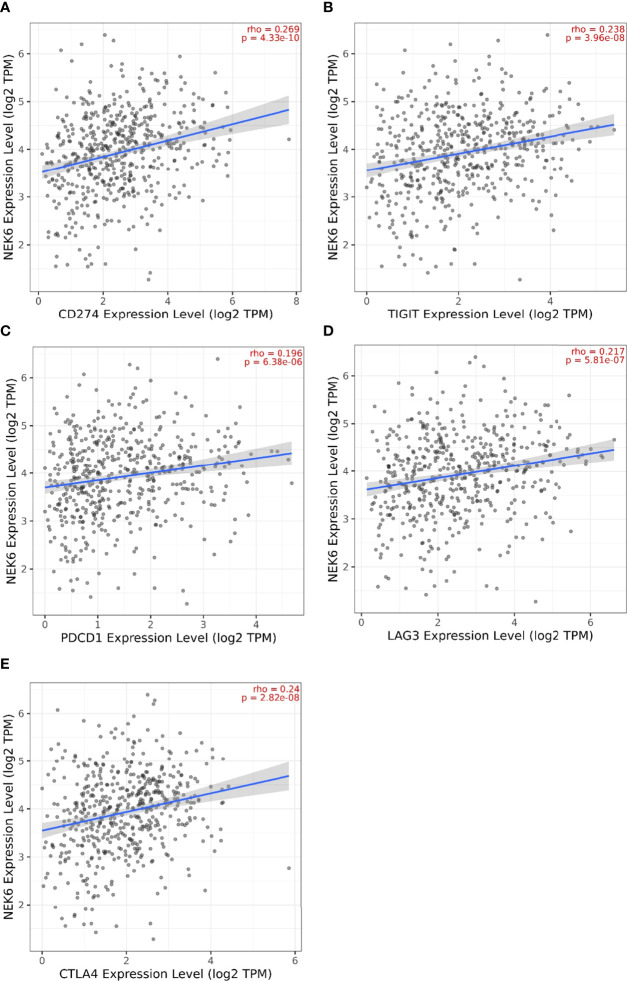
Immune checkpoint marker associated with NEK expression in HNSCC. Scatterplots of the correlations between NEK6 expression and CD274 **(A)**, TIGIT **(B)**, PDCD1 **(C)**, LAG3 **(D)** and CTLA-4 **(E)** in HNSCC using the TIMER database.

We further investigated the correlation between NEK6 expression and well-known T-cell checkpoints such as LAG3, PDCD1, CD274, TIGIT, and CTLA-4 in the TIMER database. NEK6 expression was significantly correlated with the expression of LAG3, PDCD1, CD274, TIGIT and CTLA-4 in HNSCC (**Figures 6A–E**). These findings further suggest that NEK6 plays an important role in immune escape in the HNSCC microenvironment.

## Discussion

The treatment of HNSCC remains a challenge. Some patients have poor prognosis due to insufficient clinical treatment (14–16). It is necessary to find new genes related to the occurrence and development of HNSCC to improve the survival of patients. The discovery of new targeted genes enables treatments with greater specificity and sensitivity. In particular, finding new molecular targets related to immune infiltration is crucial for improving the therapeutic effect of HNSCC. Here, we aimed to investigate the function of NEK6 gene in HNSCC and its effect on tumor immune infiltration.

In our study, NEK6 was higher in multiple types of cancer tissues than in corresponding normal tissues. We further verified the high expression of NEK6 in HNSCC by immunohistochemistry. Furthermore, survival curves showed that NEK6 expression was an independent prognostic factor in HNSCC. Therefore, NEK6 has potential diagnostic value for HNSCC. Previews researches showed in liver cancer, breast cancer and gastric cancer the high expression of NEK6 is associated with poor prognosis of patients (6, 11, 17, 18). Combined with previous studies and our findings, we speculate that high expression of NEK6 may predict poor prognosis in HNSCC patients in the real world. Our results showed that the transcript level of NEK6 was not correlated with the stage of HNSCC, suggesting NEK6 may have little relationship with cell proliferation. Its role in cancer cells may require further study.

We analyzed NEK6-related pathways in HNSCC to understand their oncogenic mechanisms. It was found that the functional network of NEK6 in HNSCC is mainly related to extracellular matrix organization, extracellular structure organization, external encapsulating structure organization, and ameboidal-type cell migration by GO and KEGG analysis. It also plays an important role in signaling pathways such as transmembrane receptor protein serine/threonine kinase signaling pathway activity, TGFβ response, and response to growth factor stimulation. KEGG pathway analysis showed that NEK6 gene was enriched in PI3K-Akt signaling pathway, focal adhesion and regulation of actin cytoskeleton. GSVA analysis showed that NEK6 was enriched in functions such as angiogenesis and epithelial mesenchymal transition. All these results suggest that NEK6 affects the degradation of extracellular matrix (19, 20), cell motility (21) and other aspects, mainly by affecting cell morphology such as EMT.

To further evaluate the potential immune mechanism of NEK6 in HNSCC, we analyzed the level of NEK6-related immune infiltration. The results showed that NEK6 was positively correlated with infiltration levels of CD8+ T cells, B cells, neutrophil cells, mucosa-associated invariant T cells (MAIT), TEM cells and Th17 cells. NEK6 was negatively correlated with DCs, monocyte cells, nTreg cells, Macrophage, Tr1, CD4 T cells, iTreg cells, NKTs. Some of these infiltrating cells contradict the function of NEK6, such as CD8+ T cells, mucosa-associated invariant T cells (MAIT), and Th17 cell infiltration is positively correlated with NEK6 expression. Previous studies have shown that these cells inhibit cell invasion and metastasis in cancer (22–25). Therefore, the relationship between NEK6 expression and immune cell infiltration deserves to be further investigated in future work.

Most importantly, we investigated the correlation of NEK6 and immune checkpoint expression, including LAG3, PDCD1, CD274, TIGIT and CTLA-4, which correlate with response to ICB. These immune checkpoints are highly expressed in HNSCC (24, 26, 27). Furthermore, we found that NEK6 was positively co-expressed with these immune checkpoints, which may partially explain the cancer-promoting role of NEK6 by analyzing the correlation between immune checkpoints and NEK6.

Our study shows that immune cell infiltration recruited by NEK6 has both positive and negative effects on tumor patients. The mechanism of how NEK6 affects immune cell infiltration remains unclear. A limitation of this study is the large number of samples required to validate our results. In addition, underlying immune mechanisms should be explored and NEK6 investigated as a biomarker for predicting immune response rates in real-world HNSCC patients.

## Conclusion

High expression of NEK6 in HNSCC predicts poor prognosis of patients, and NEK6 may play an important role in extracellular matrix degradation and cell motility. NEK6 alters clinical outcomes in patients with HNSCC by affecting molecular expression of immune checkpoints, and it may serve as a predictor of ICI therapy.

## Data Availability Statement

The original contributions presented in the study are included in the article/supplementary material. Further inquiries can be directed to the corresponding author.

## Ethics Statement

The studies involving human participants were reviewed and approved by the Medical Ethics Committee of Xiangya Hospital, Central South University (Hunan, China). The patients/participants provided their written informed consent to participate in this study.

## Author Contributions

W-MW, Z-MY, and TS contributed to conception and design of the study. BL, S-SY organized the database. W-MW, Z-MY, BL performed the statistical analysis. BL, Z-MY wrote the first draft of the manuscript. S-SY, BL, JZ, and W-MW wrote sections of the manuscript. All authors contributed to manuscript revision, read, and approved the submitted version. All authors contributed to the article and approved the submitted version.

## Funding

This study was funded by the National Natural Science Foundation of China (grant no.81702708, 81873717, 82170973), Natural Science Foundation of Hunan (grant no. 2018JJ3862), Open Research Fund Program of Hubei-MOST KLOS & KLOBME.

## Conflict of Interest

The authors declare that the research was conducted in the absence of any commercial or financial relationships that could be construed as a potential conflict of interest.

## Publisher’s Note

All claims expressed in this article are solely those of the authors and do not necessarily represent those of their affiliated organizations, or those of the publisher, the editors and the reviewers. Any product that may be evaluated in this article, or claim that may be made by its manufacturer, is not guaranteed or endorsed by the publisher.
