# Supplementary figures and images for: Predictive Role of NEK6 in Prognosis and Immune Infiltration in Head and Neck Squamous Cell Carcinoma
Source: Front Endocrinol (Lausanne). 2022 Jul 11;13:943686. doi: 10.3389/fendo.2022.943686 (PMC9309547; doi:10.3389/fendo.2022.943686)

D

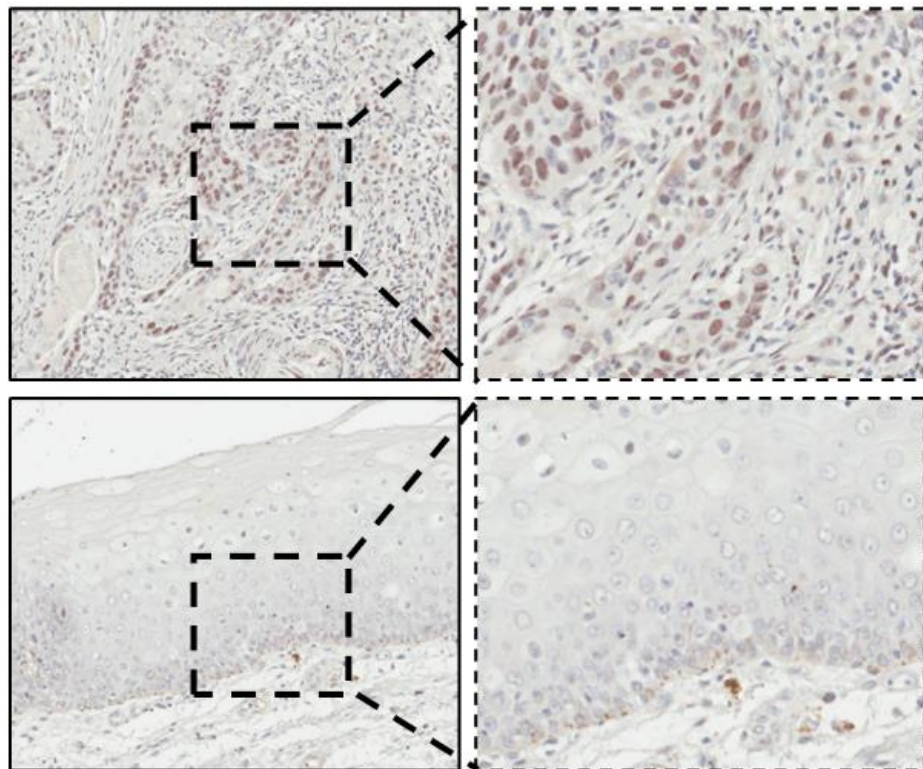

Picture in article

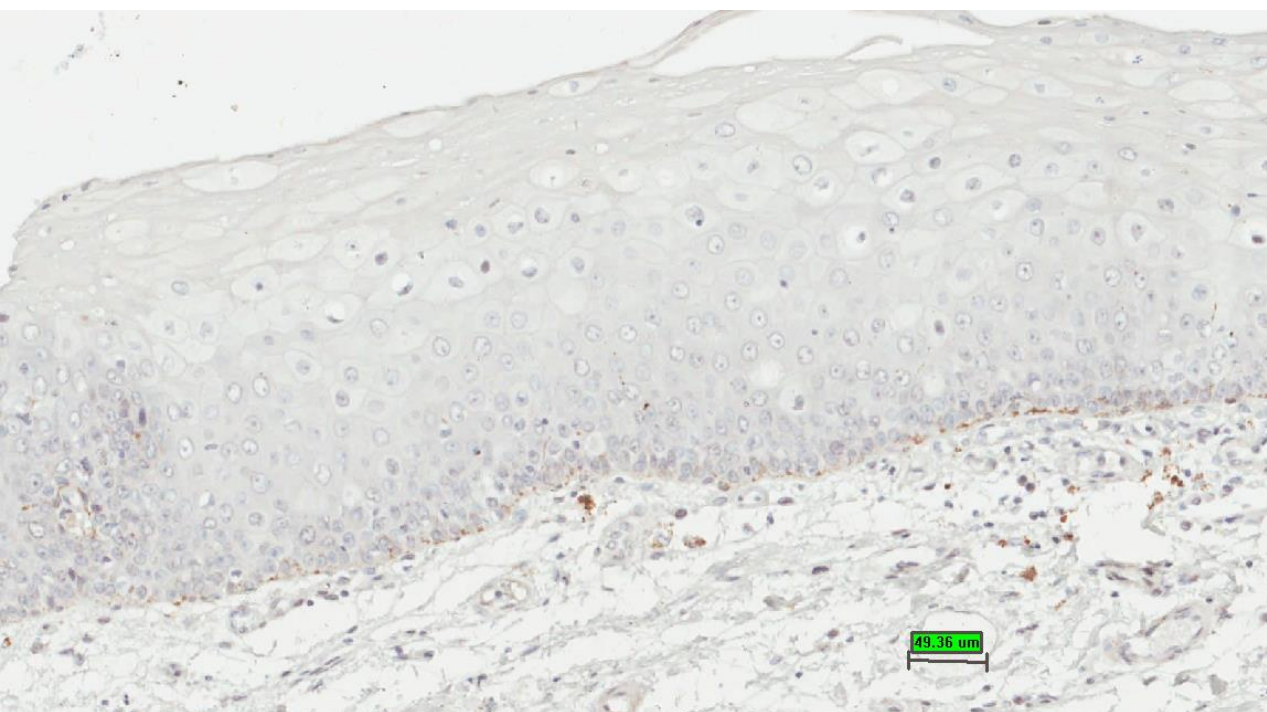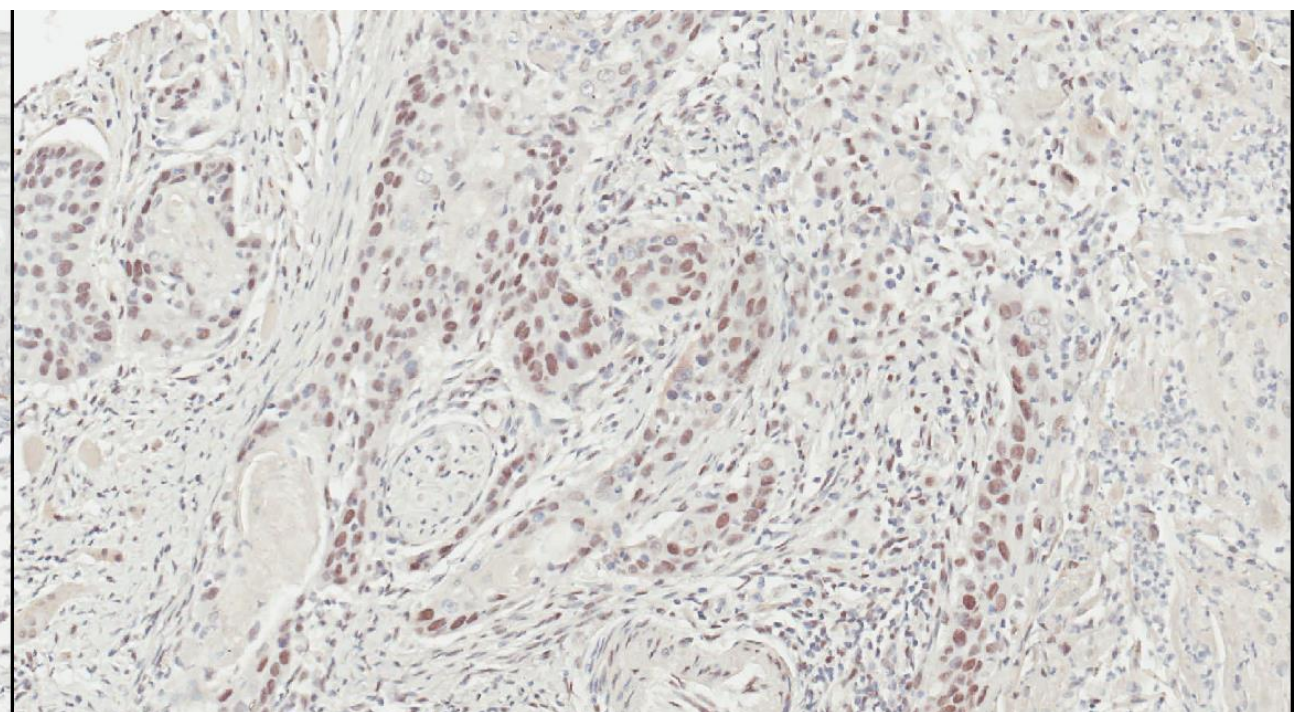

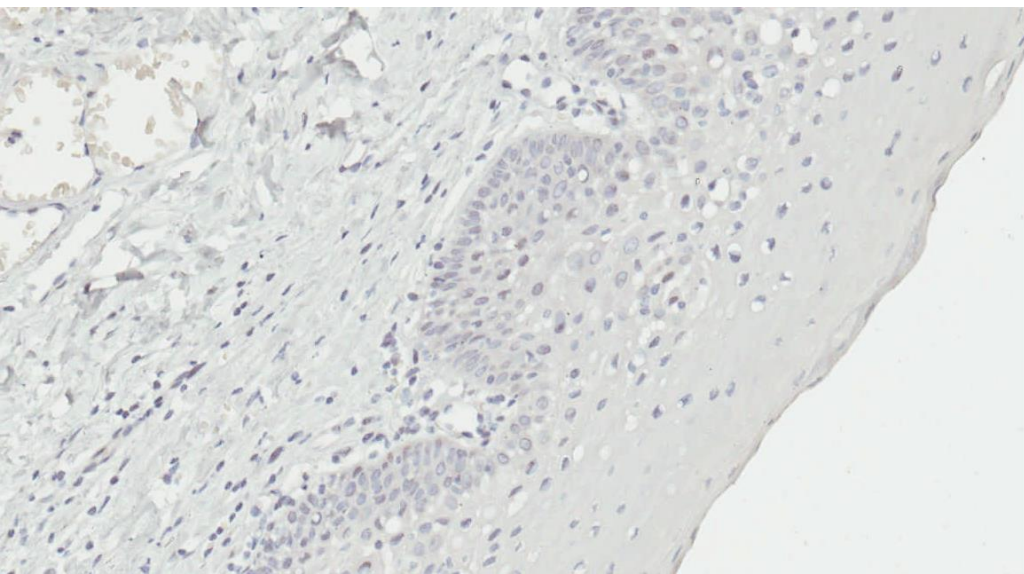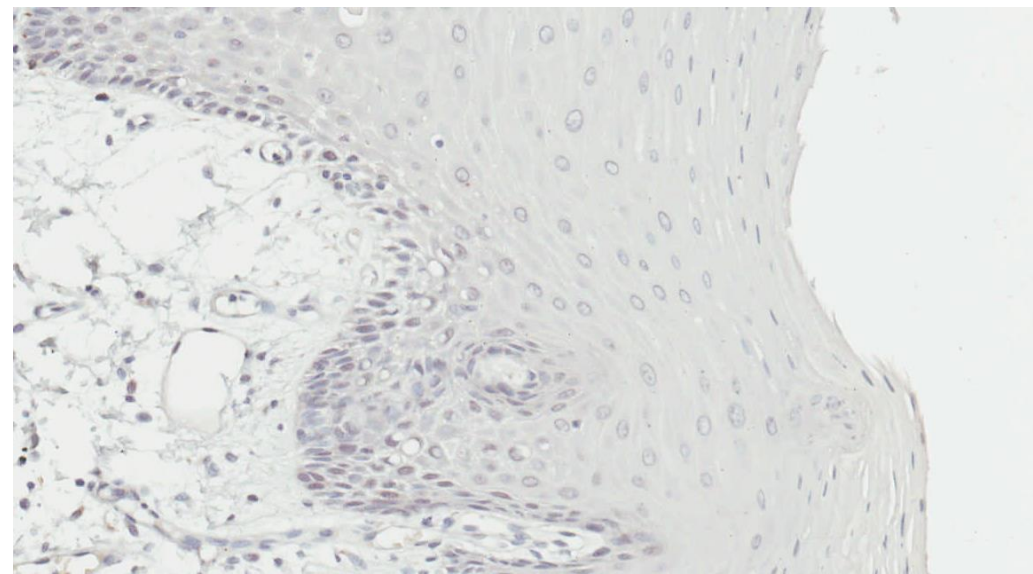

NM

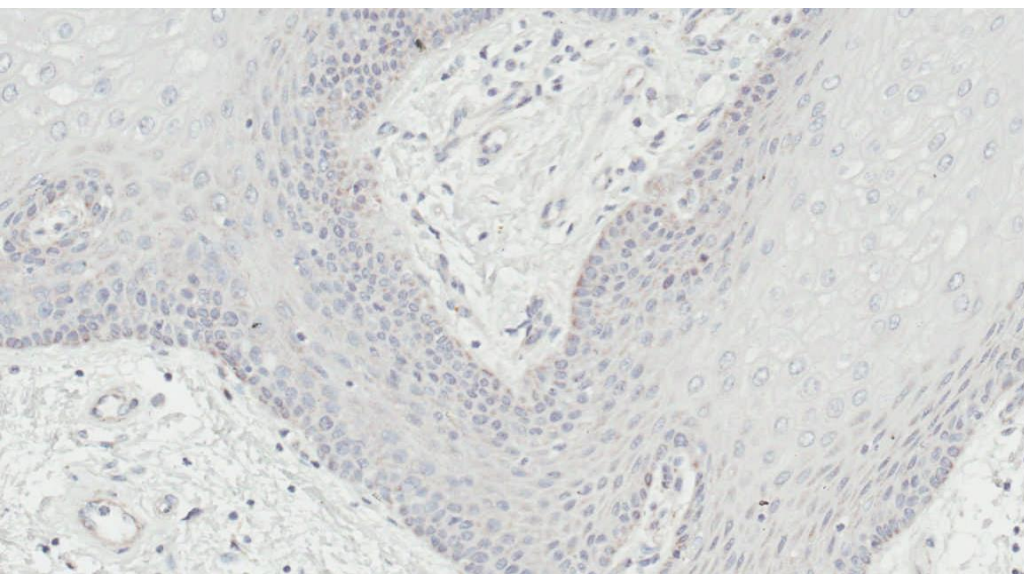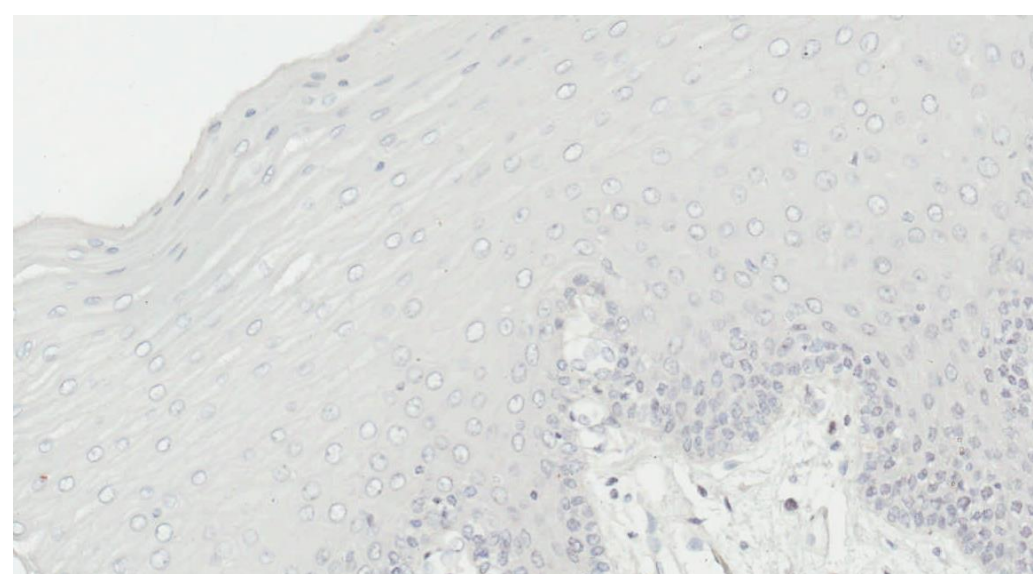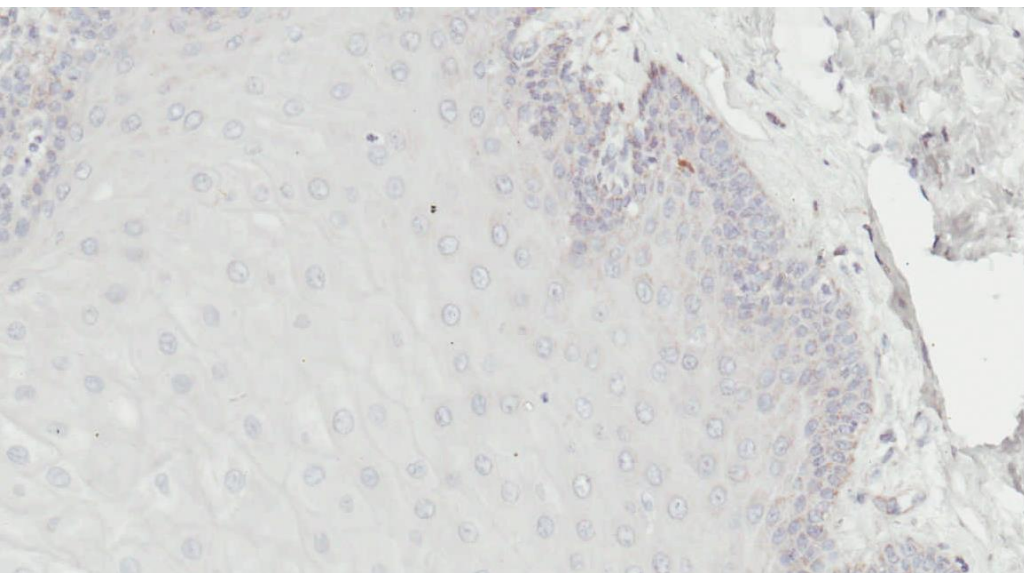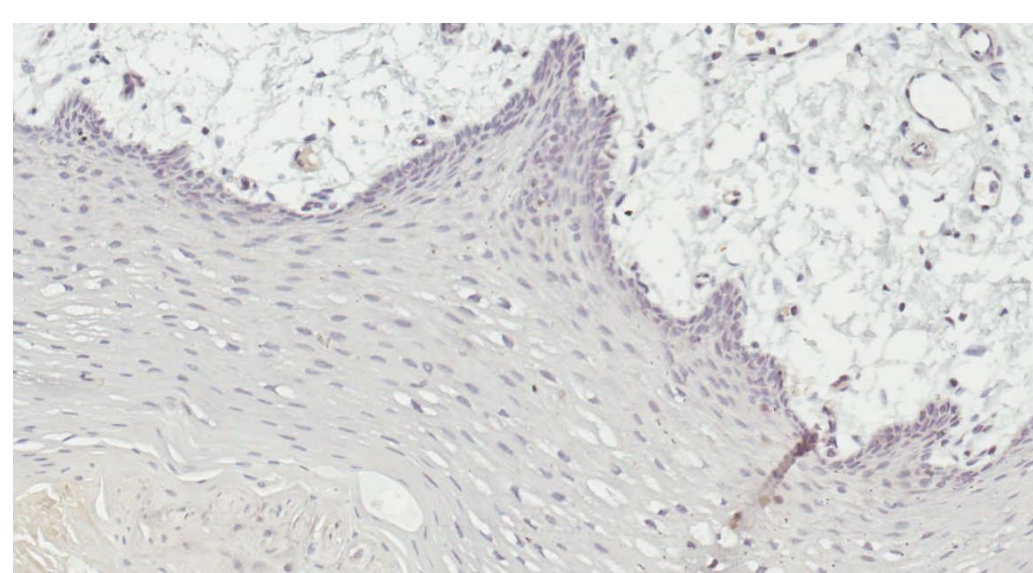

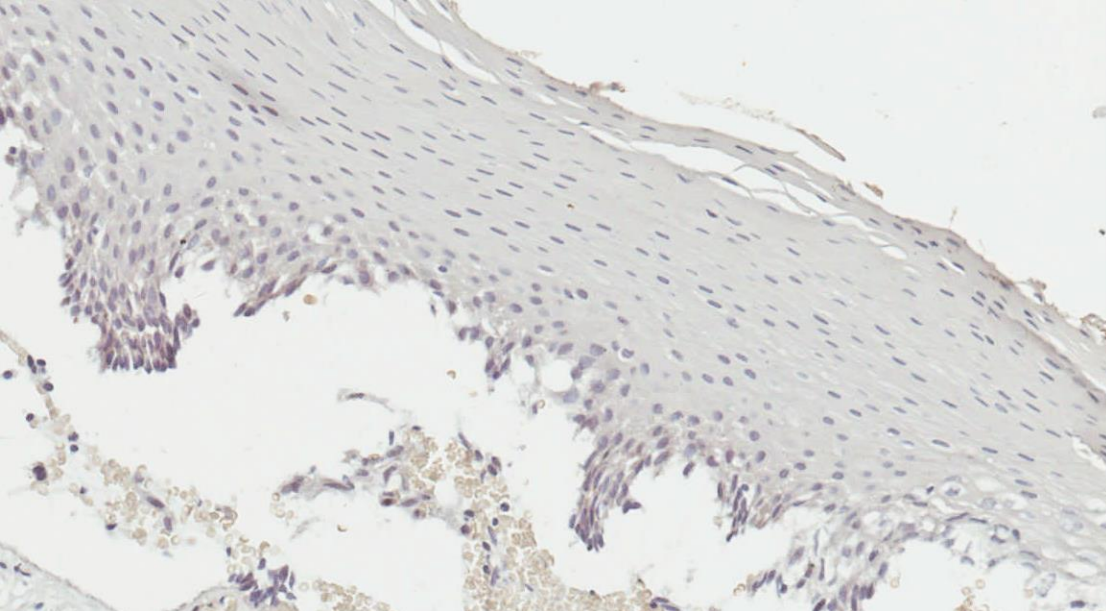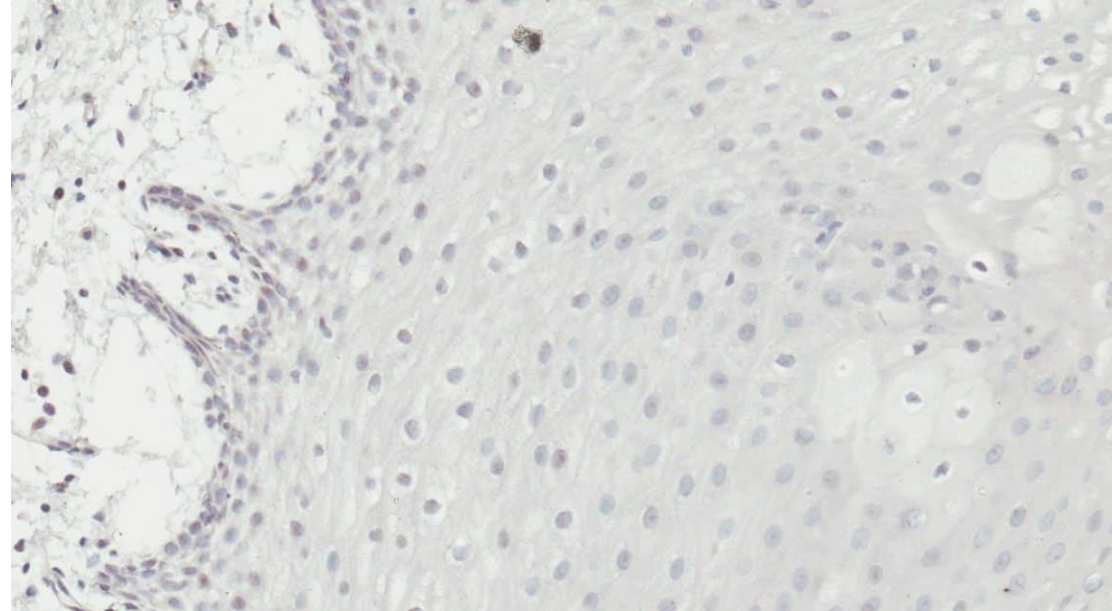

NM

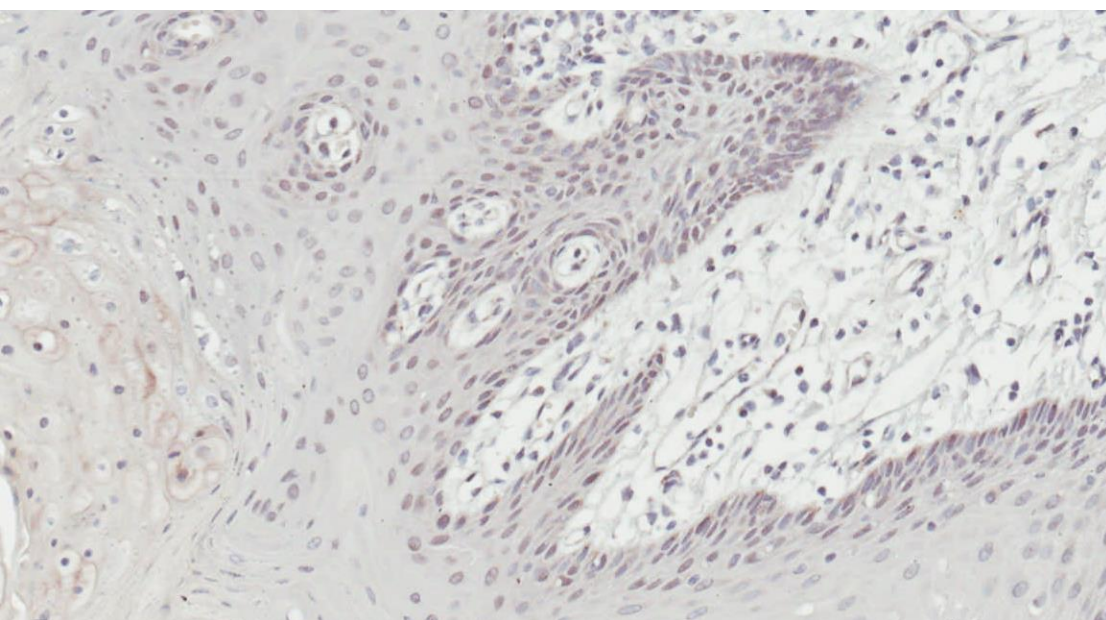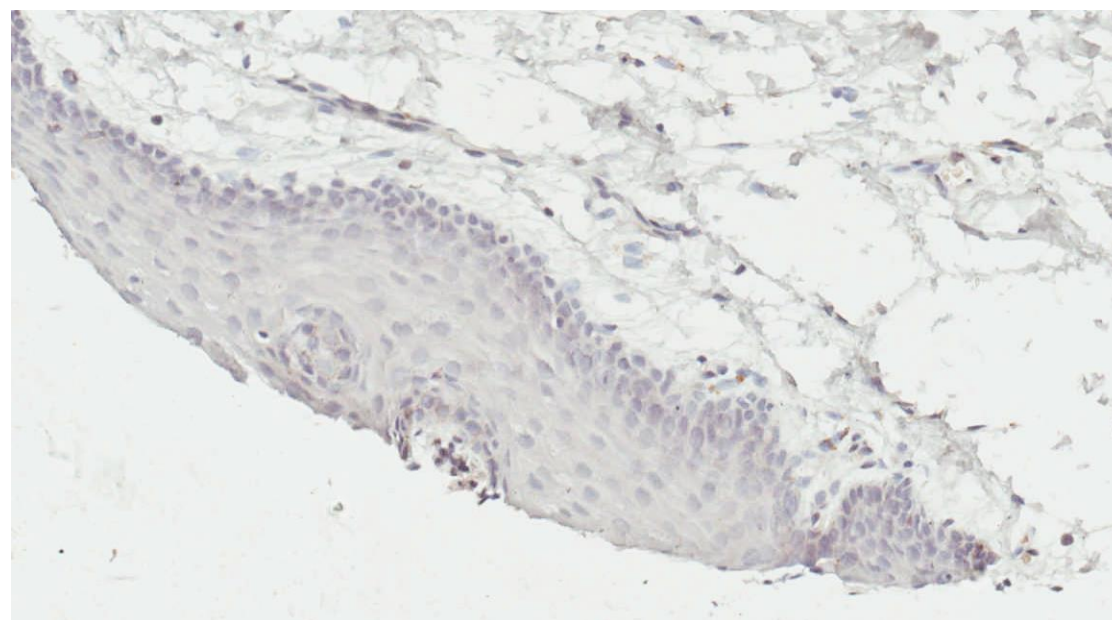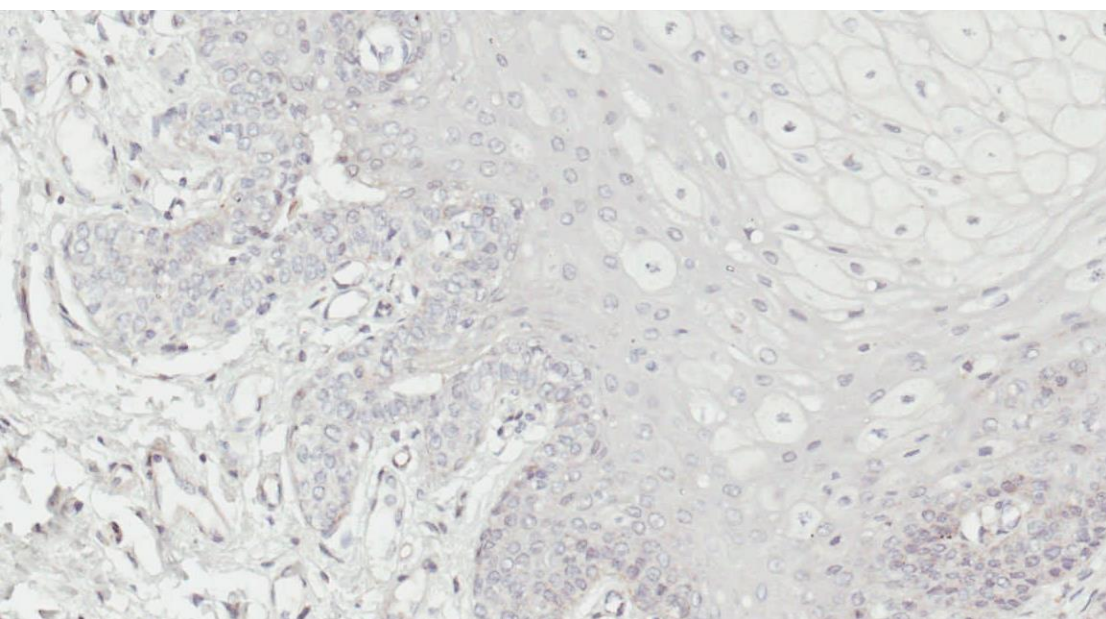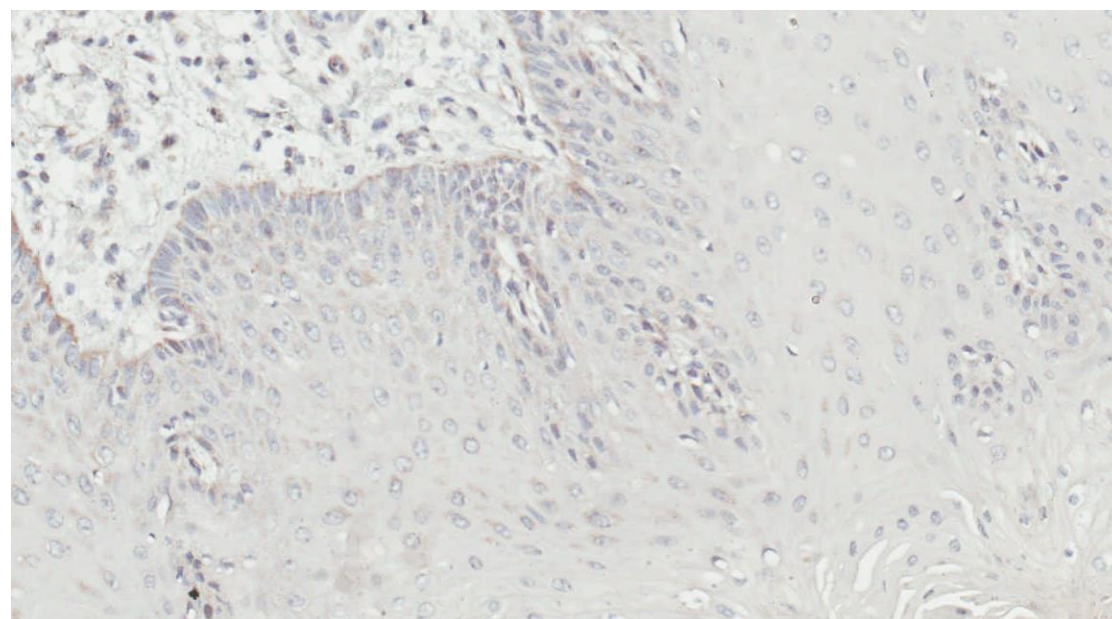

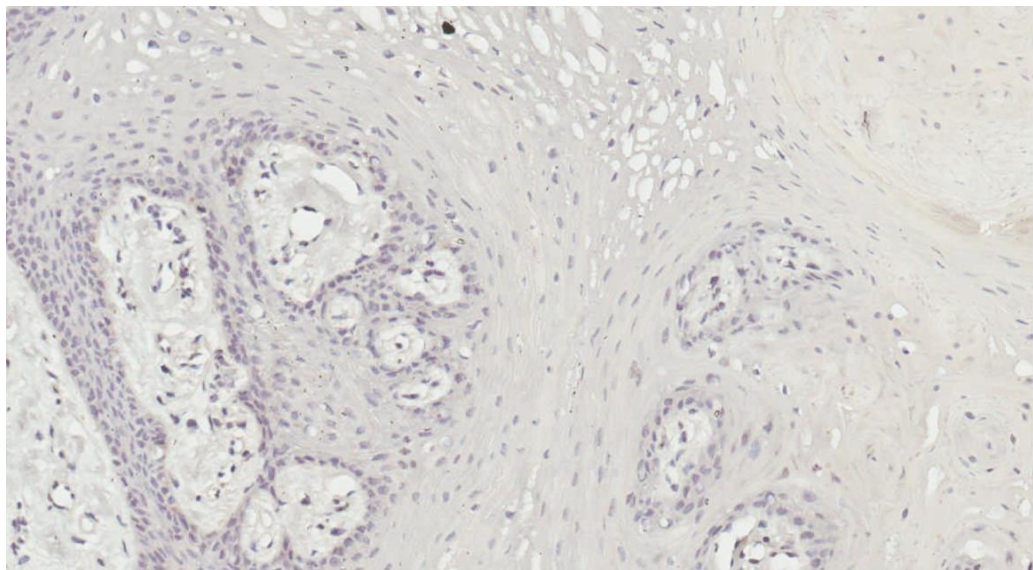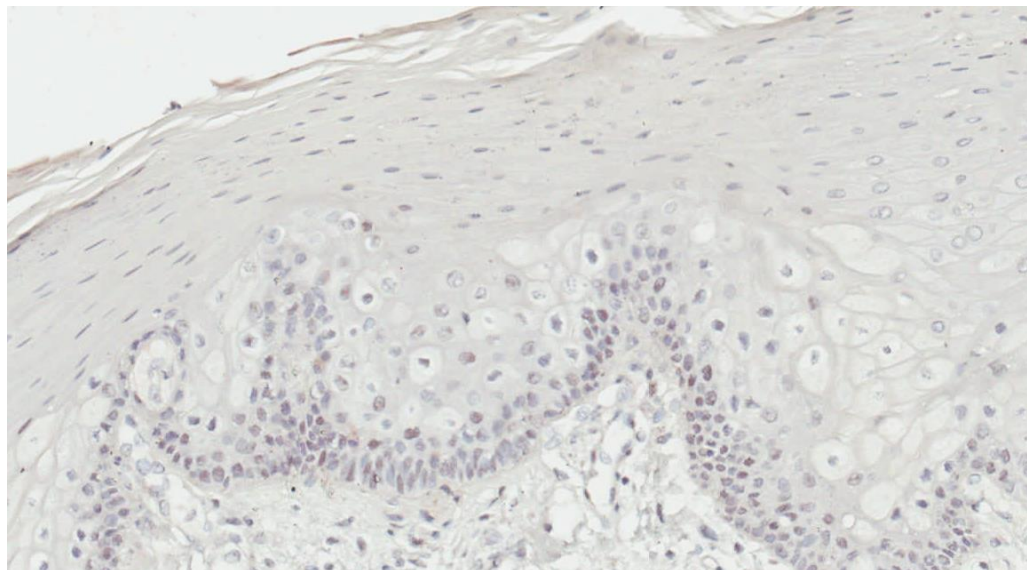

NM

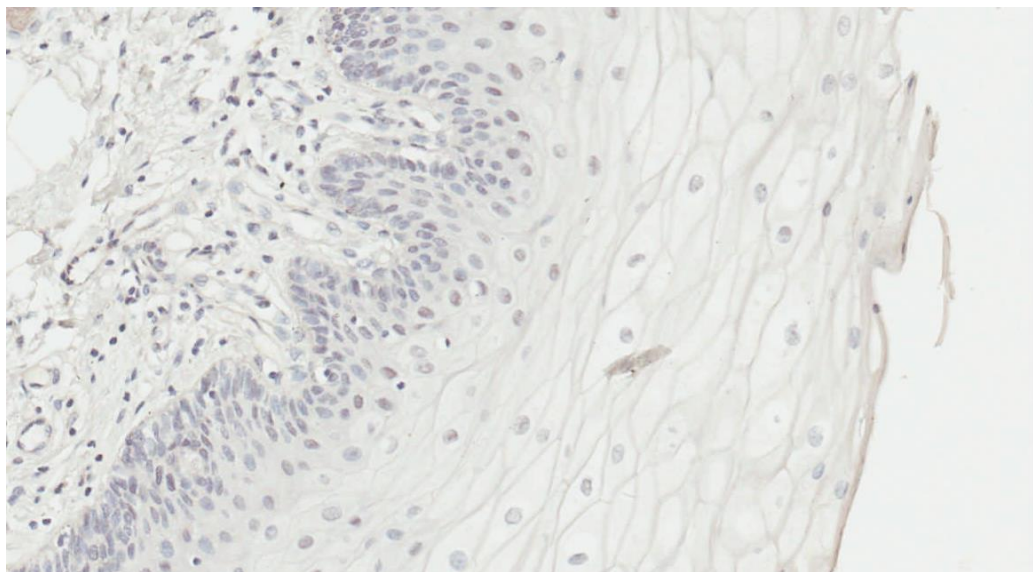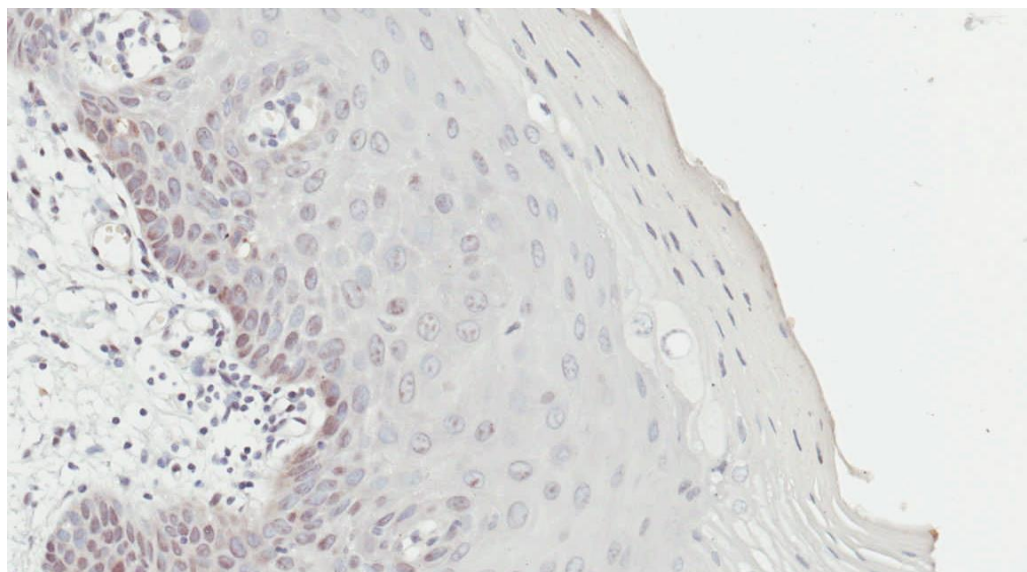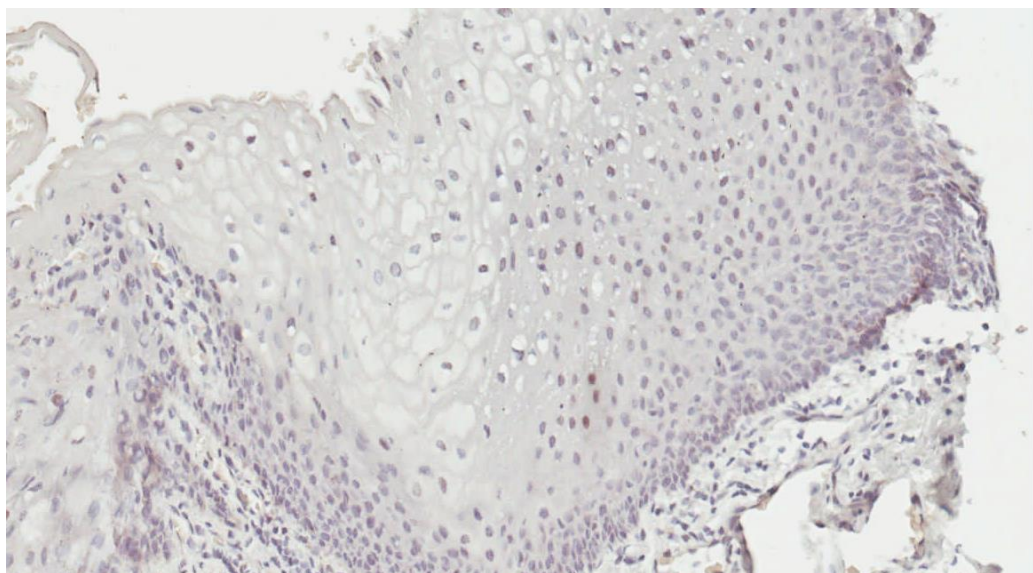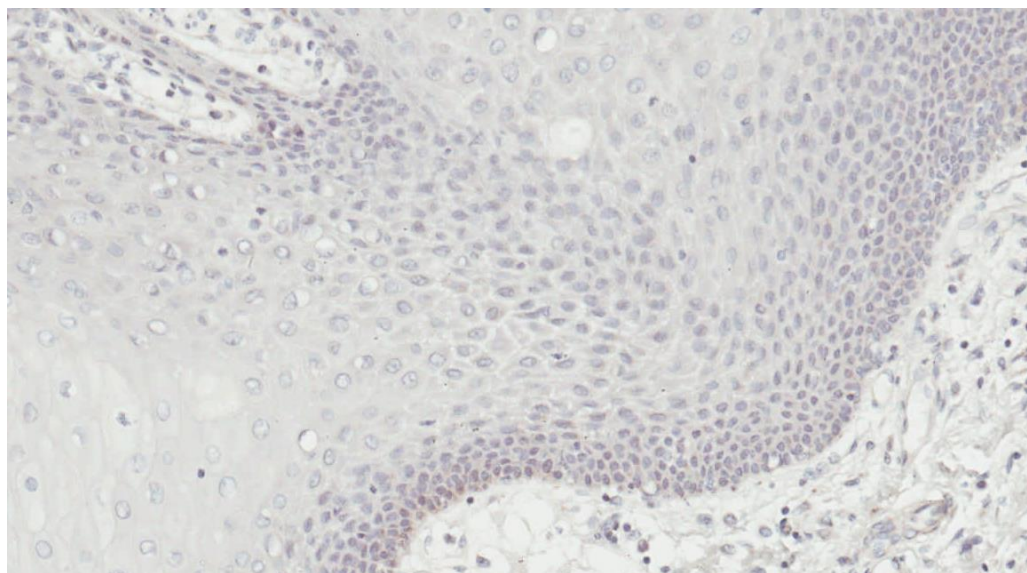

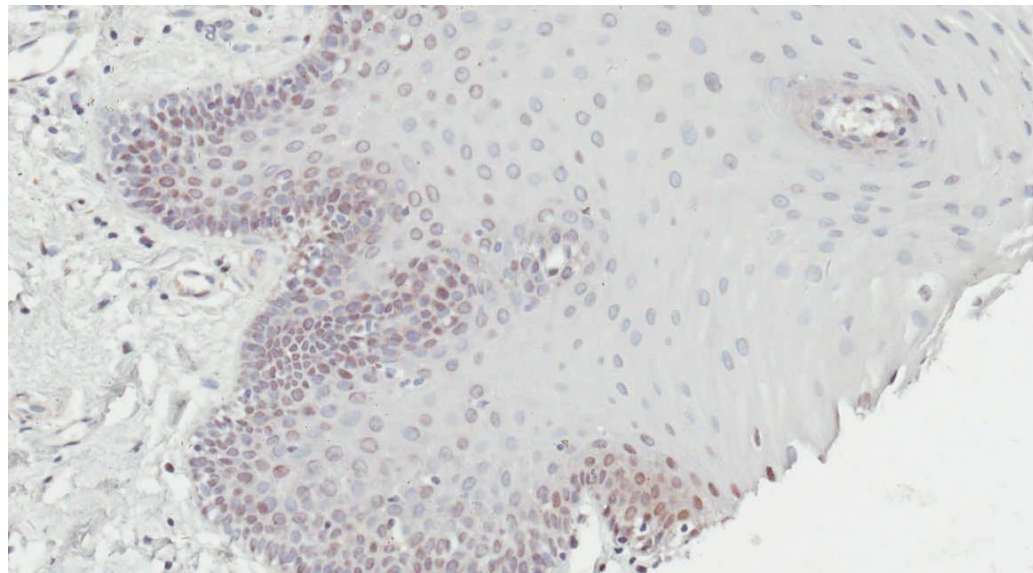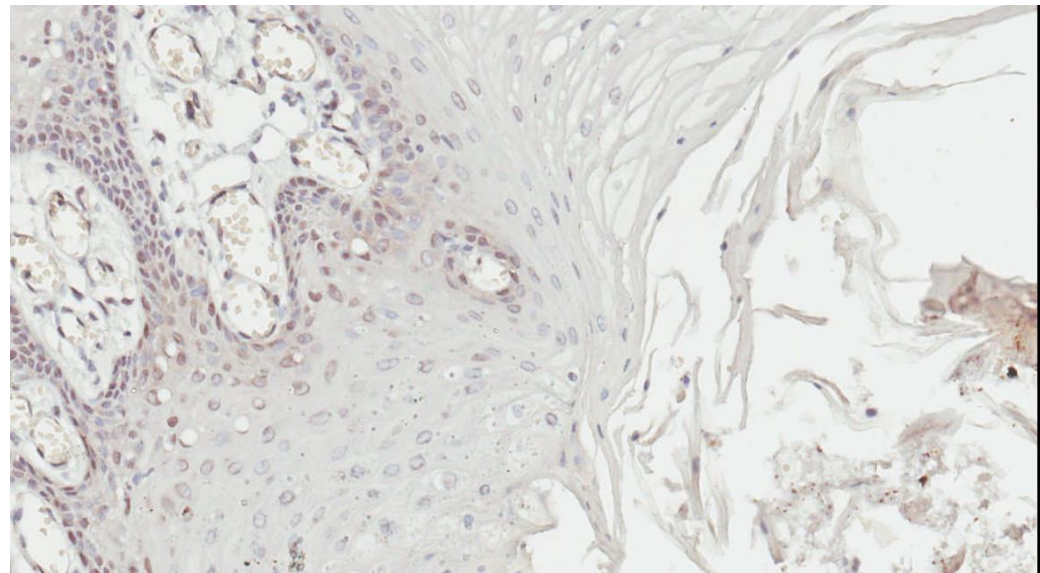

NM

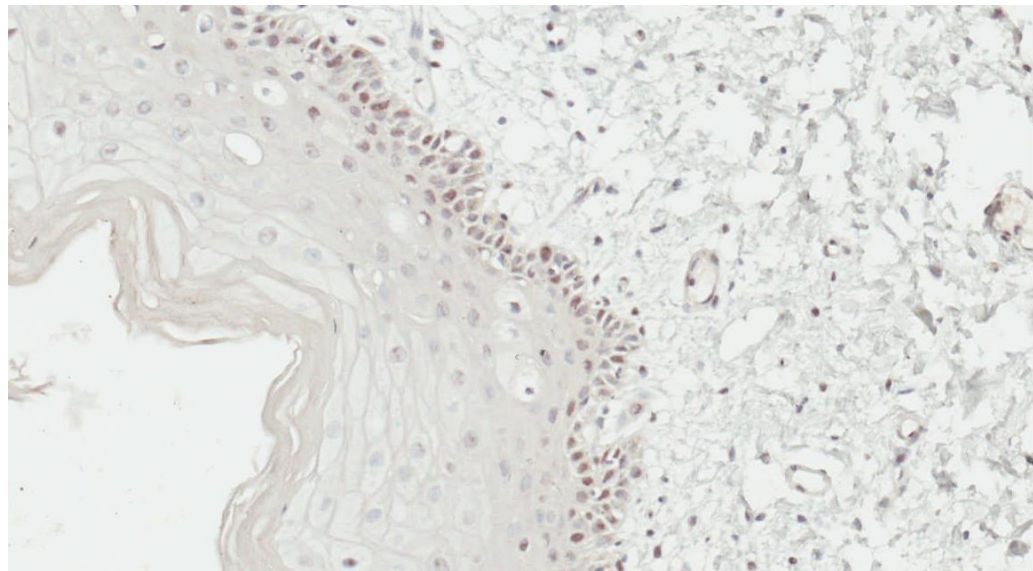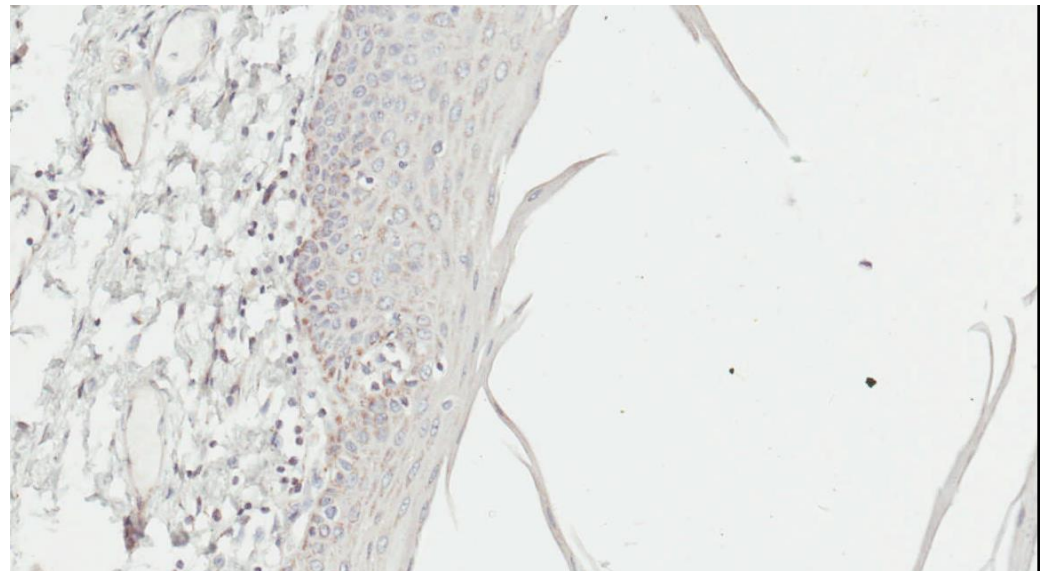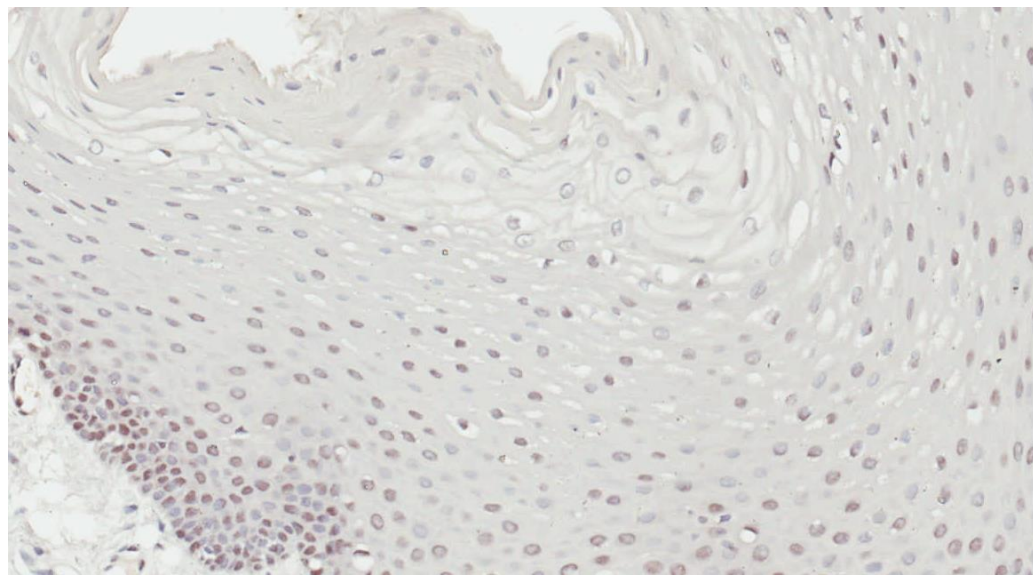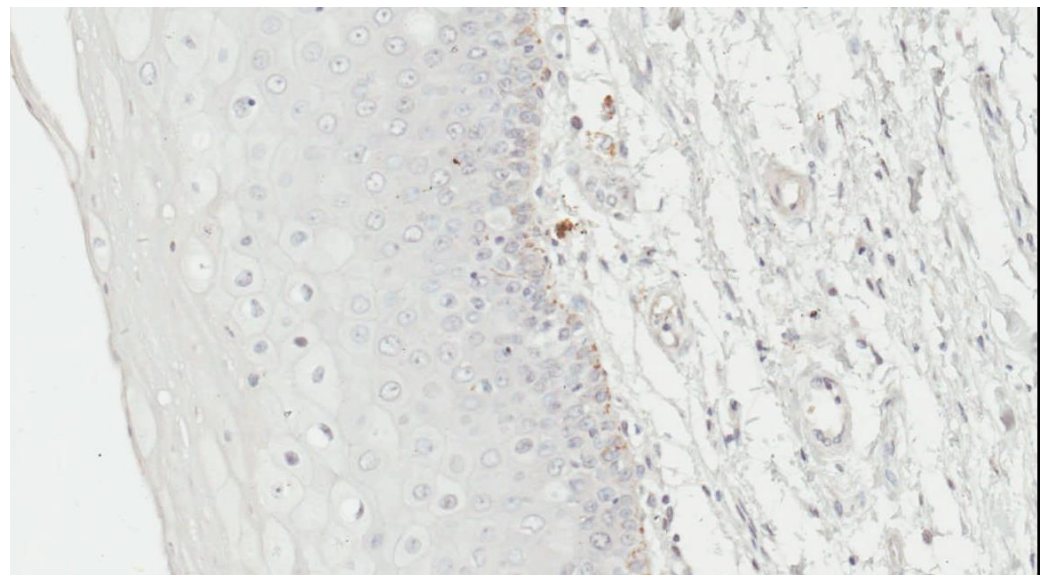

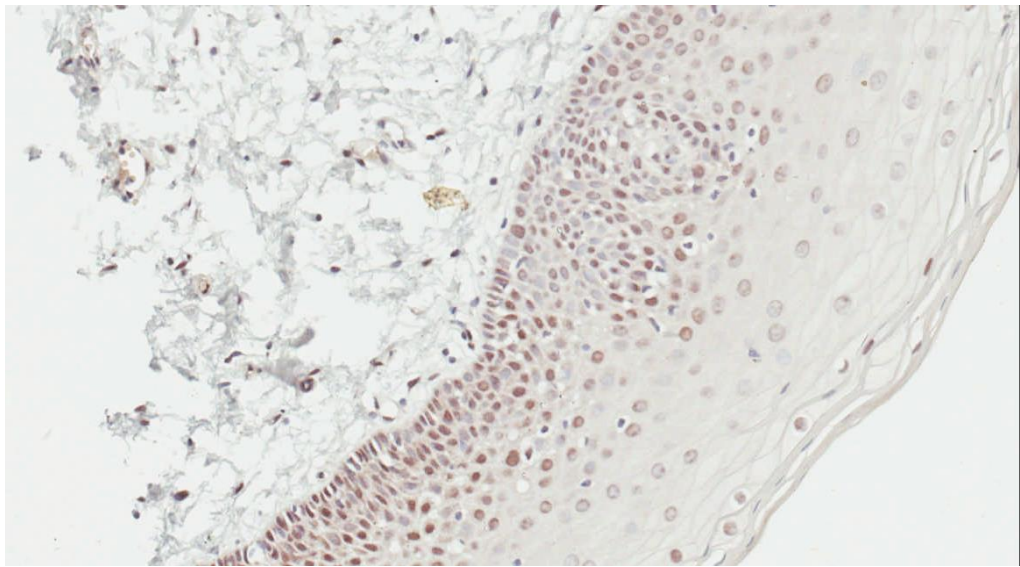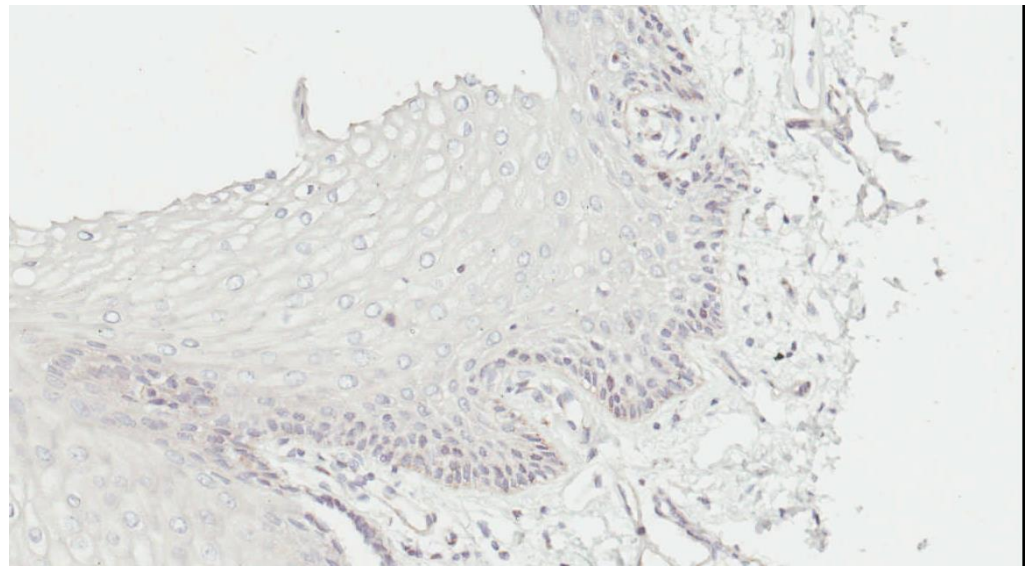

NM

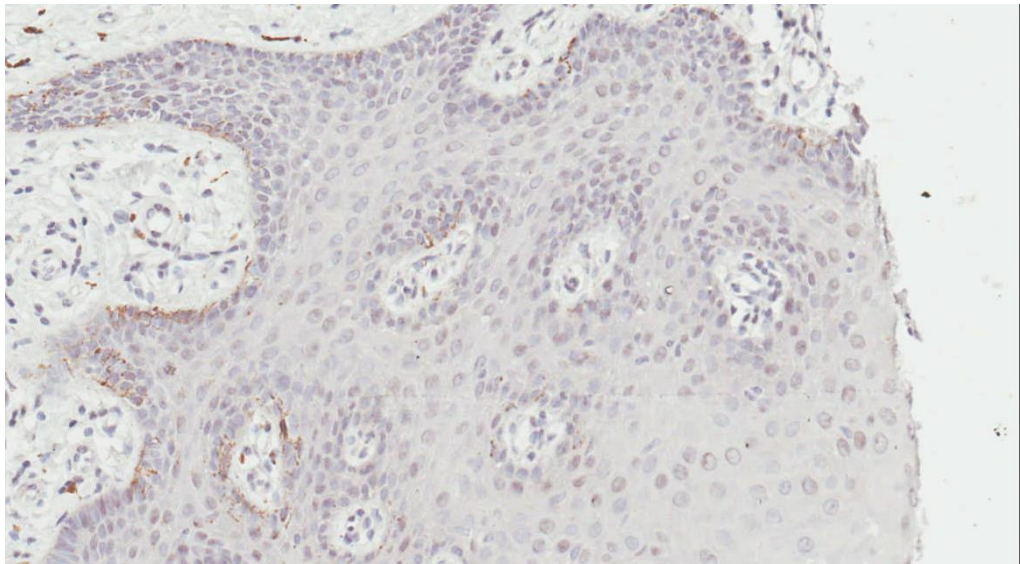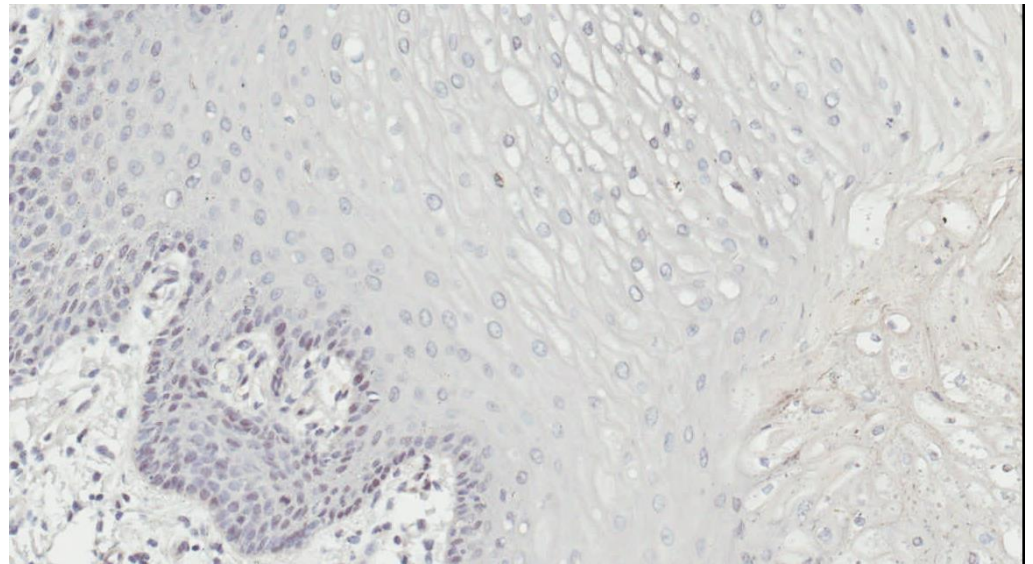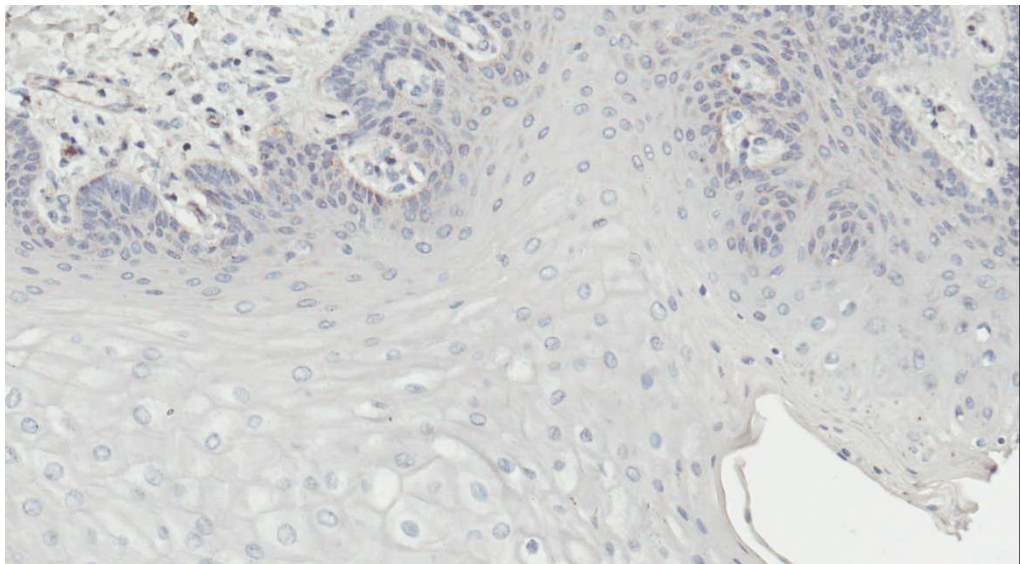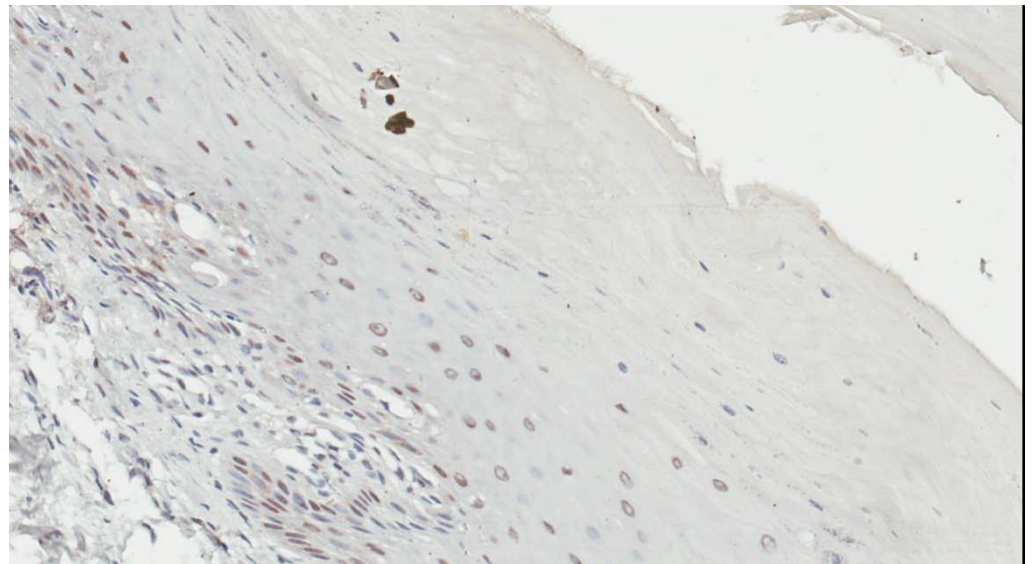

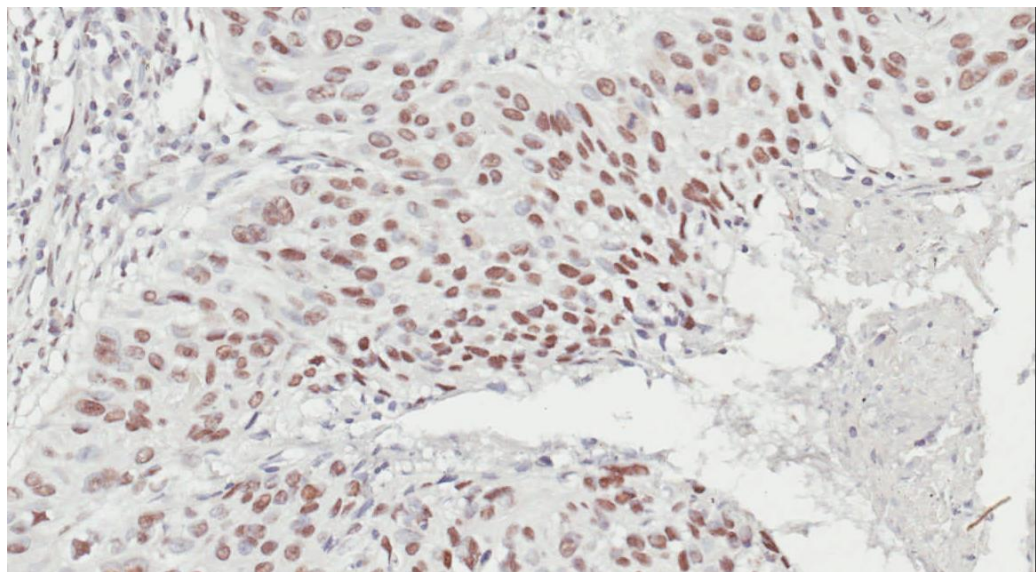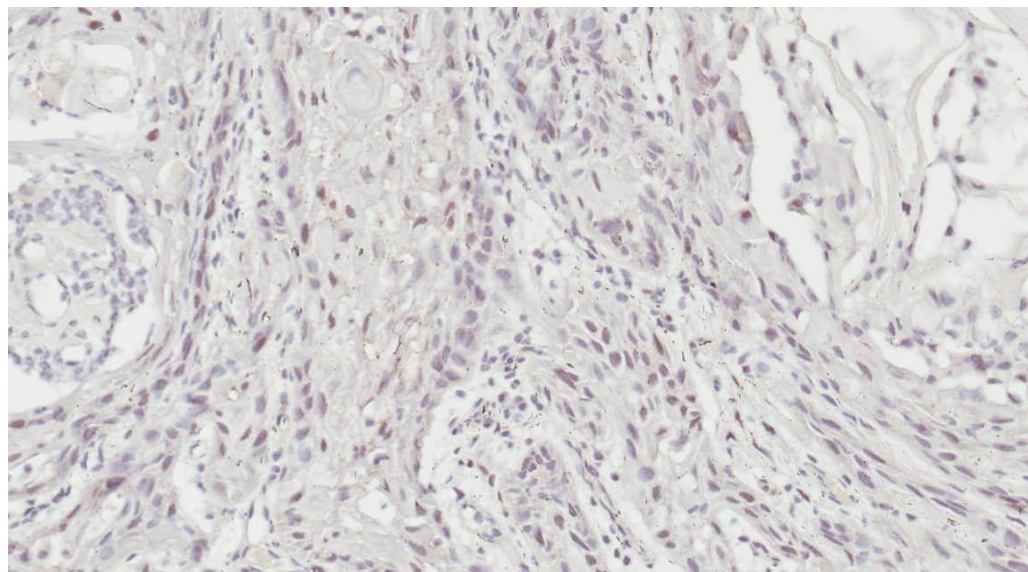

HNSC

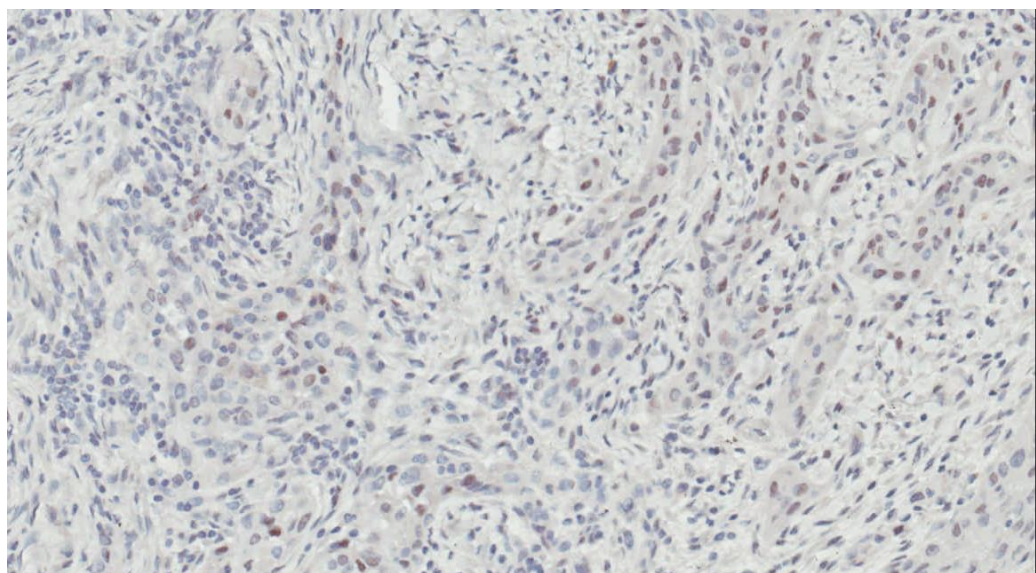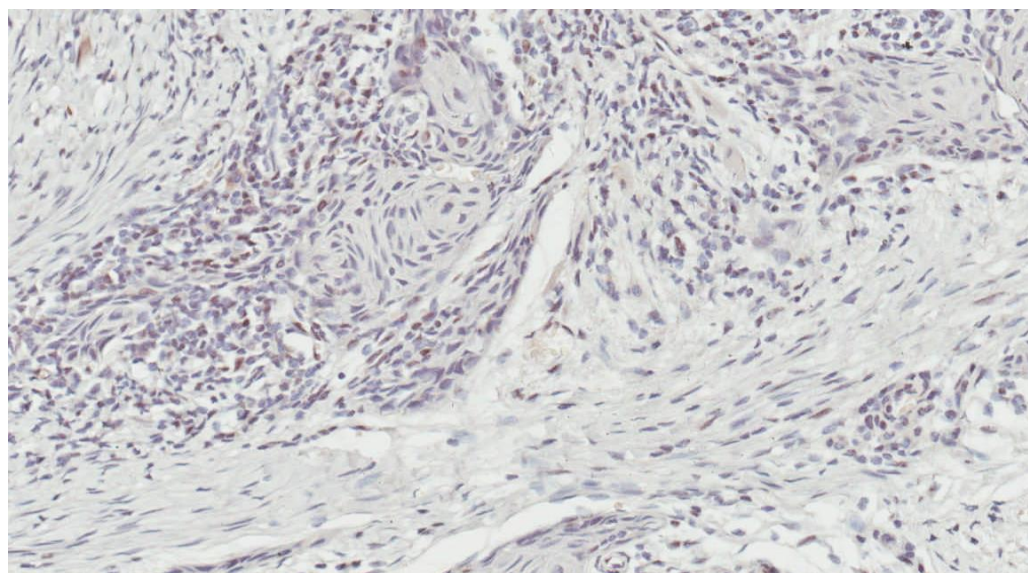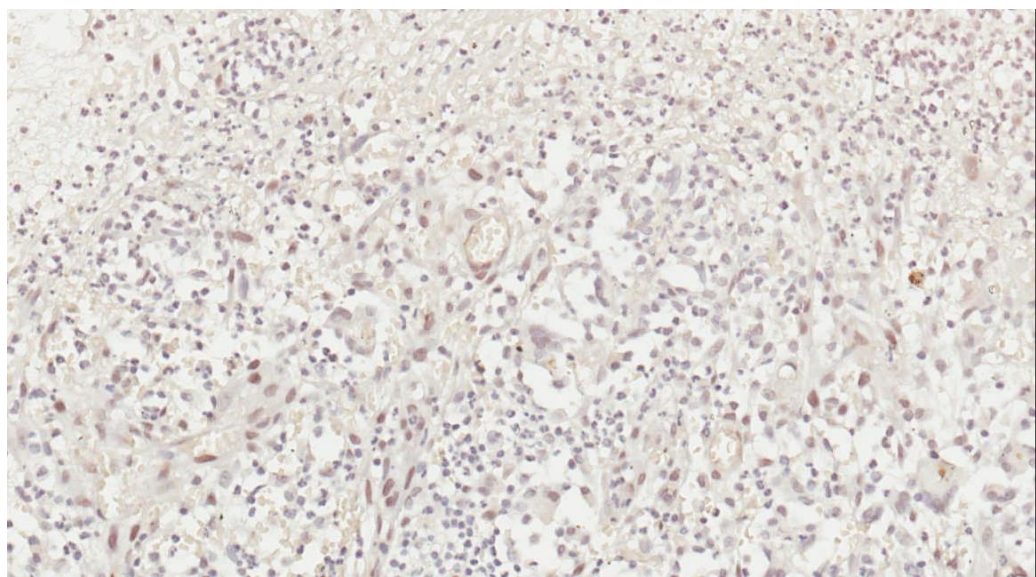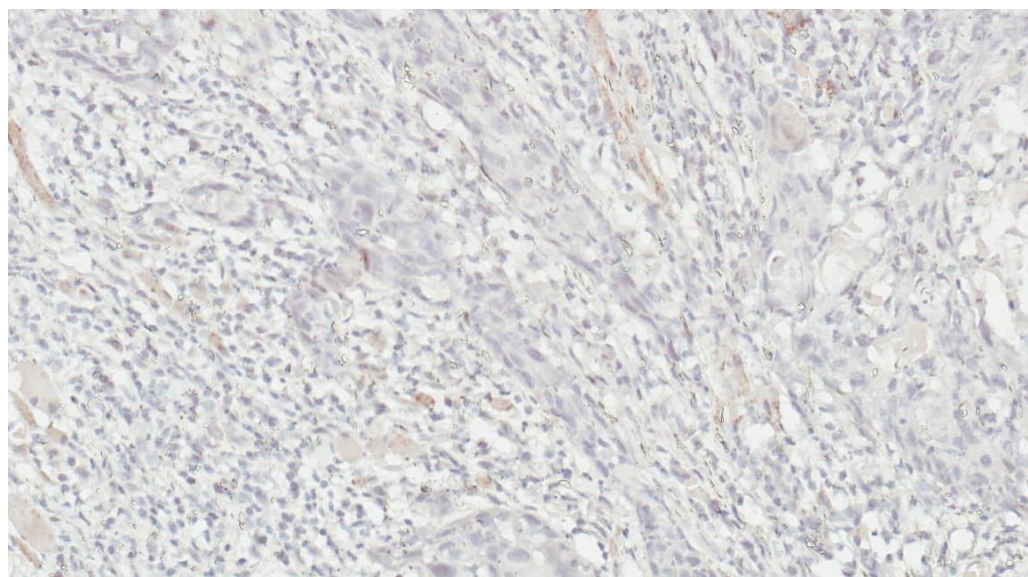

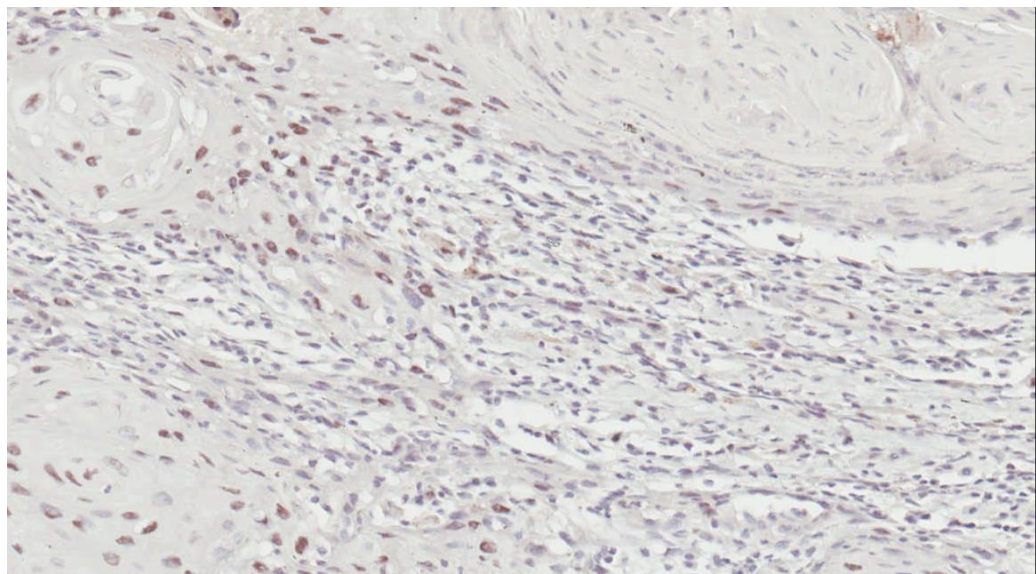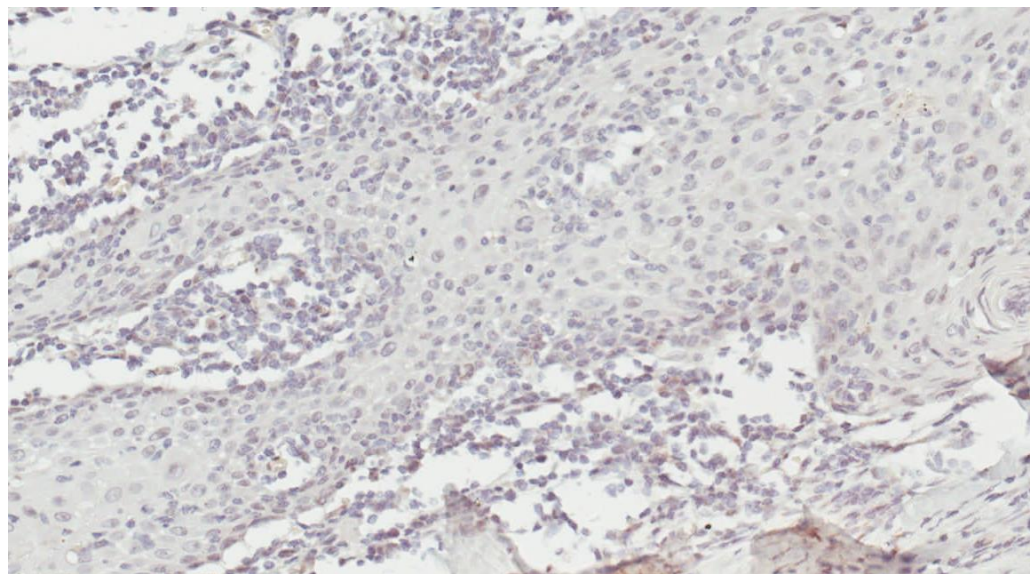

HNSC

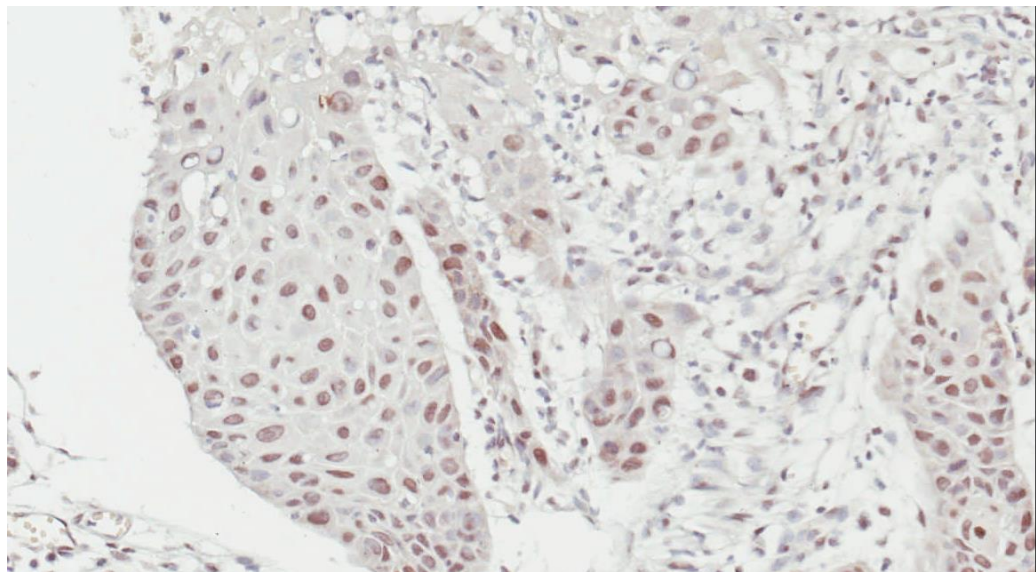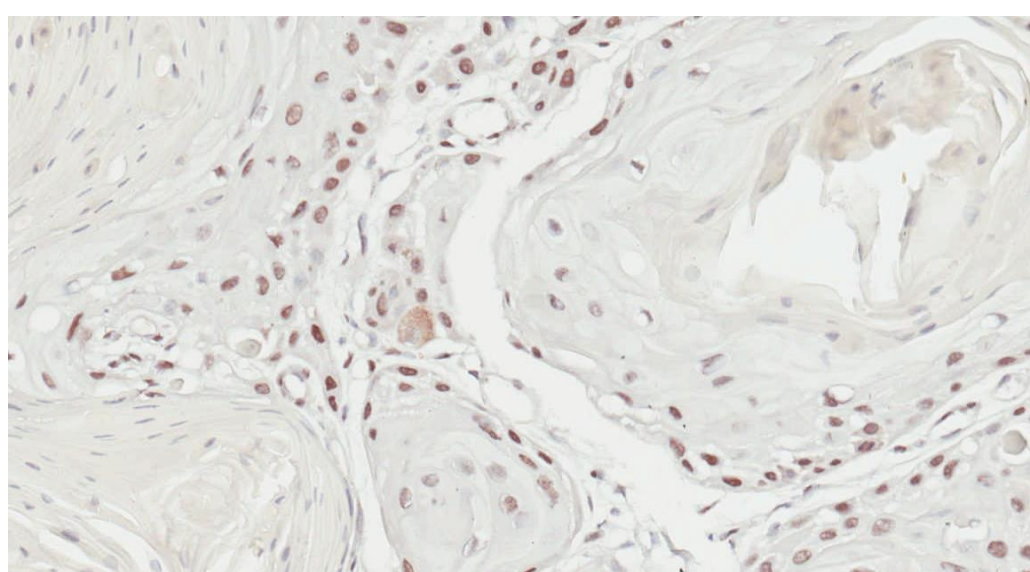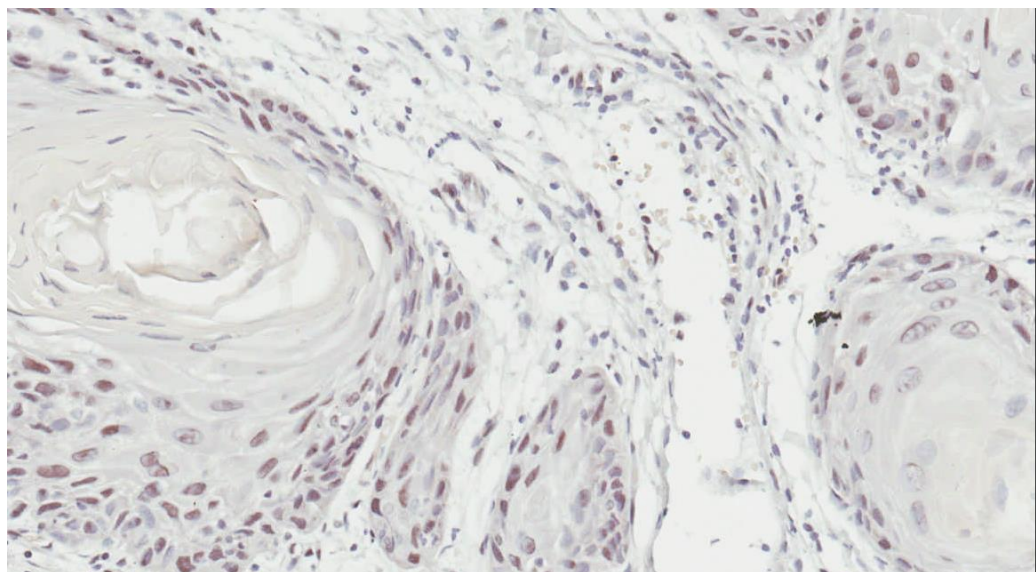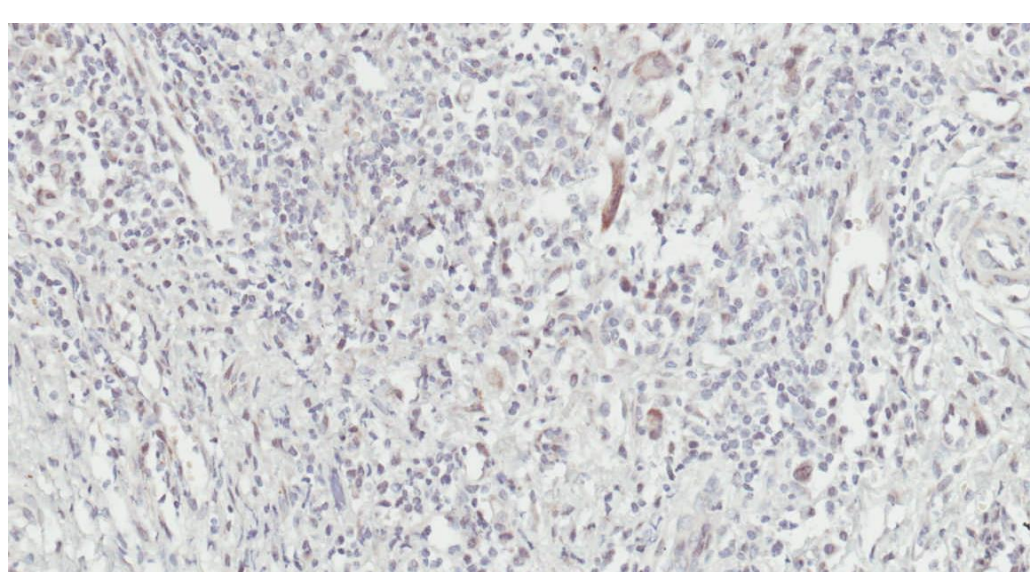

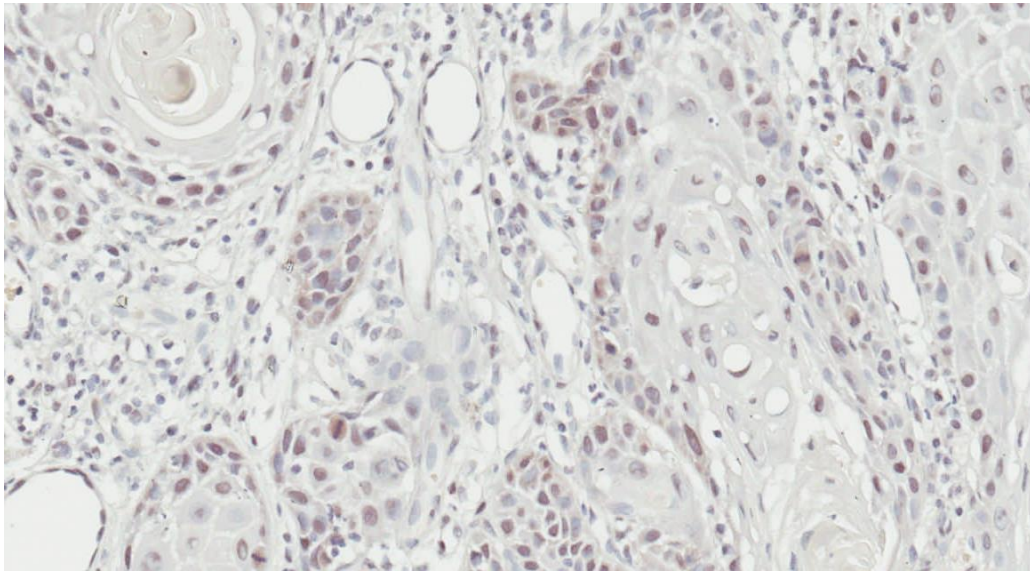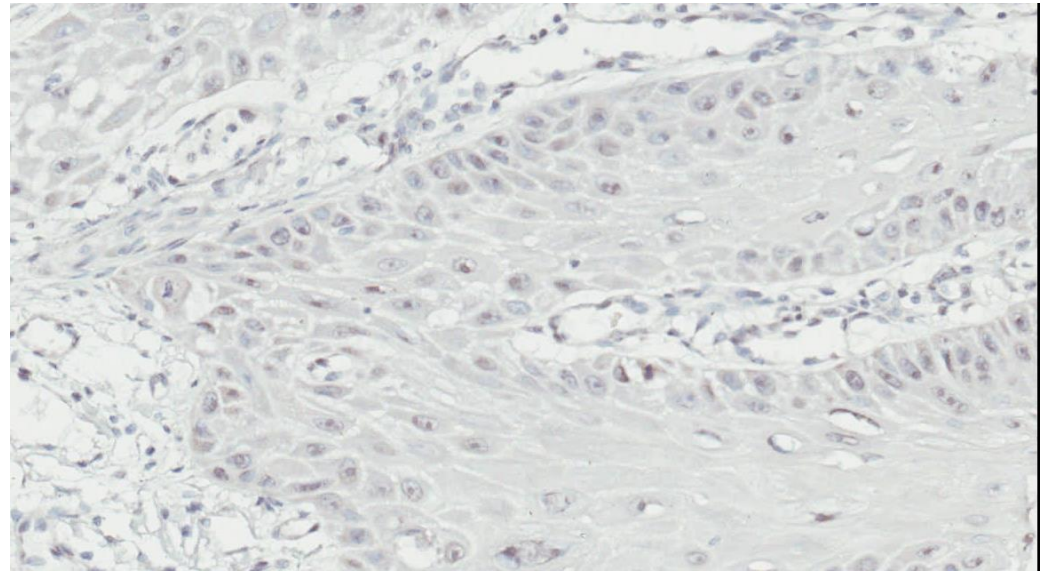

HNSC

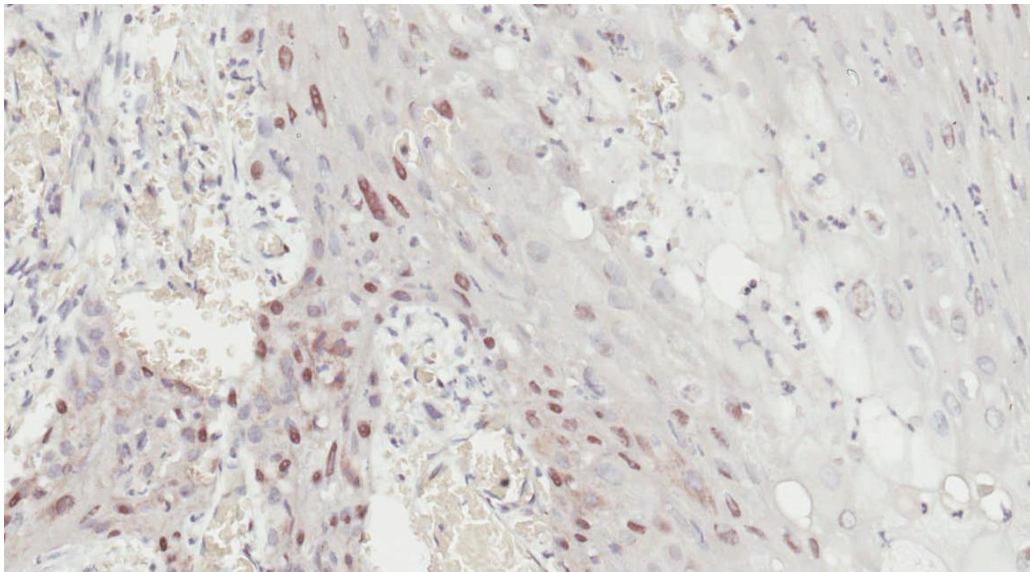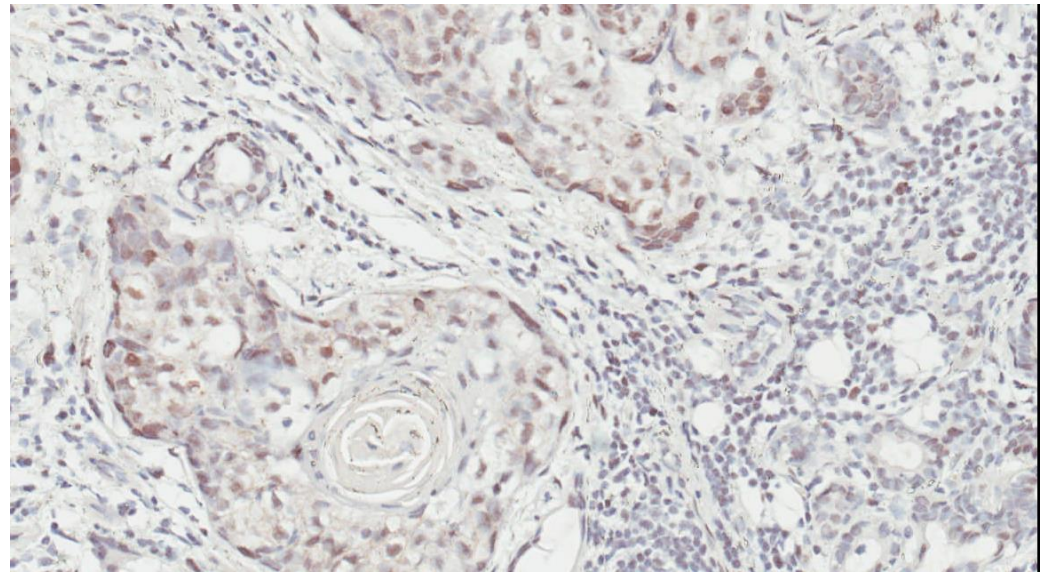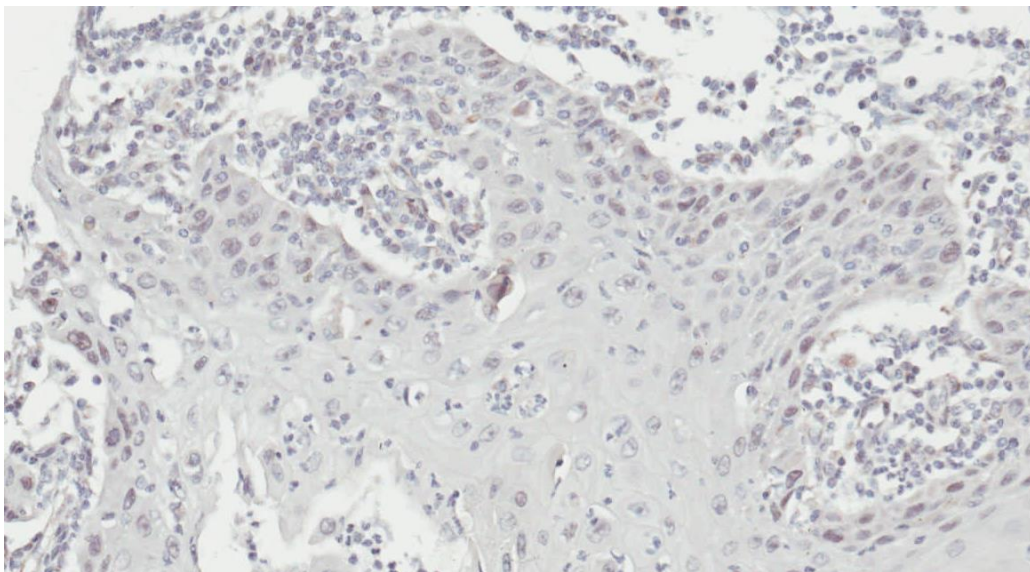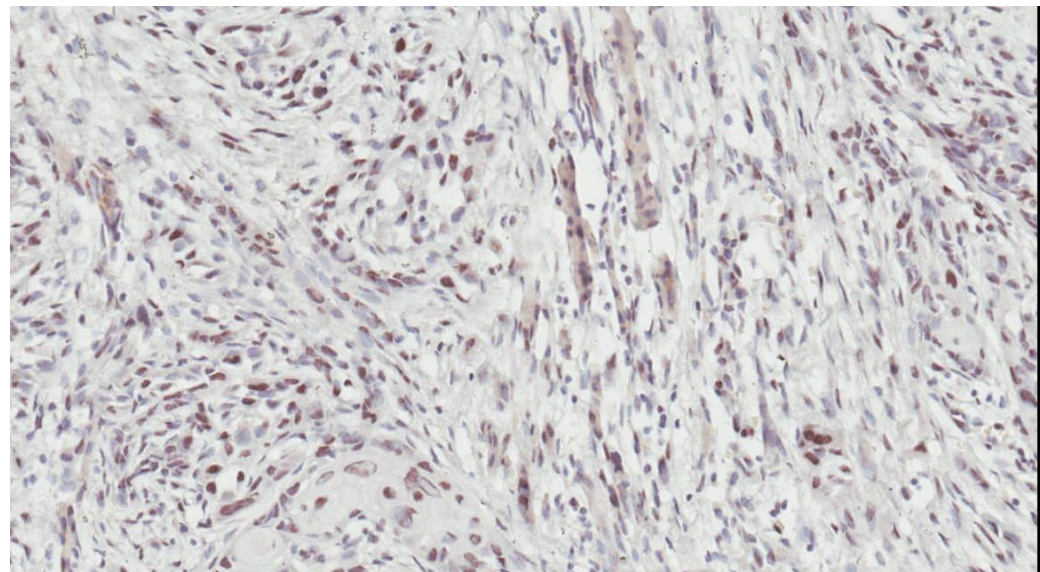

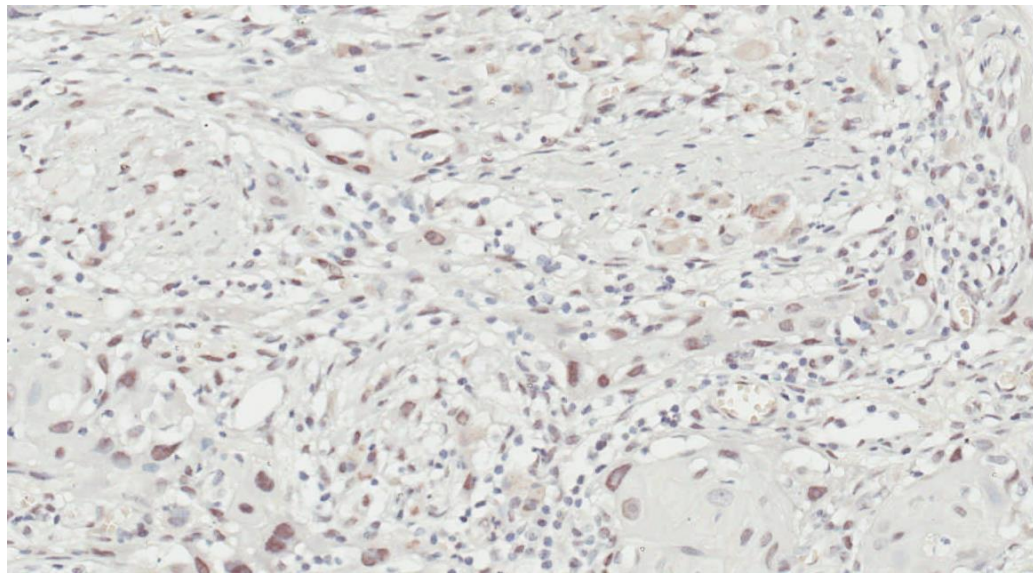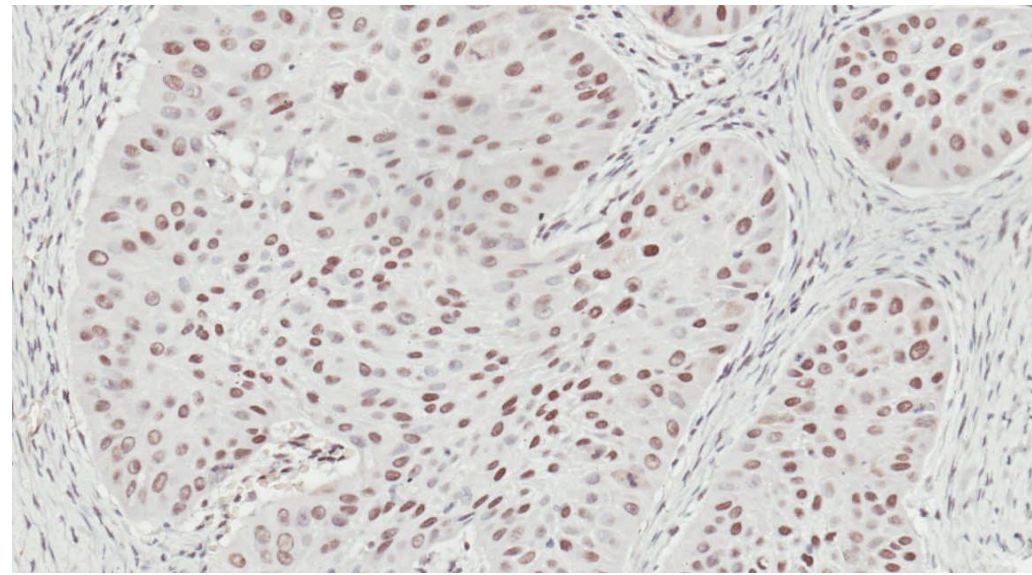

HNSC

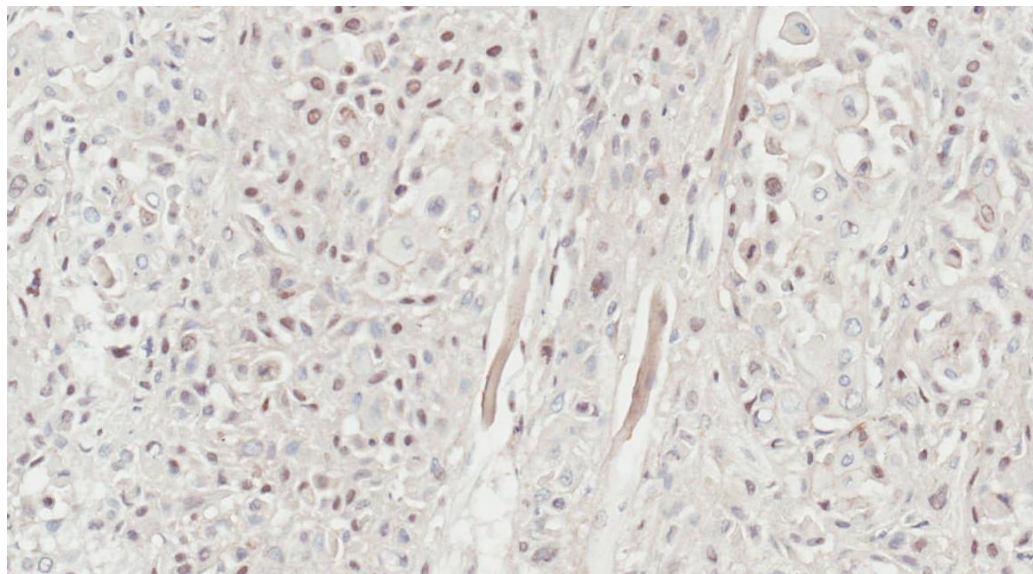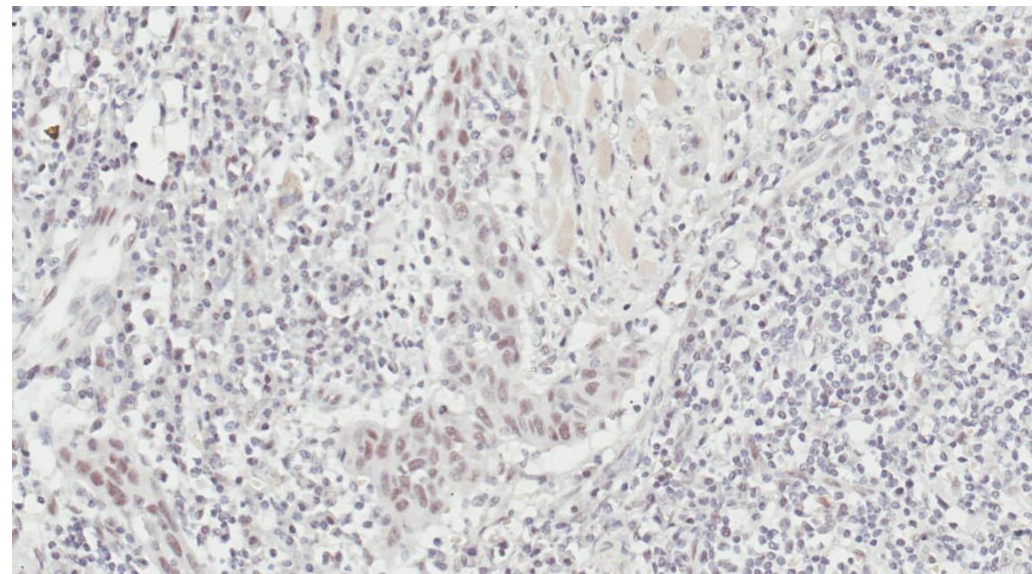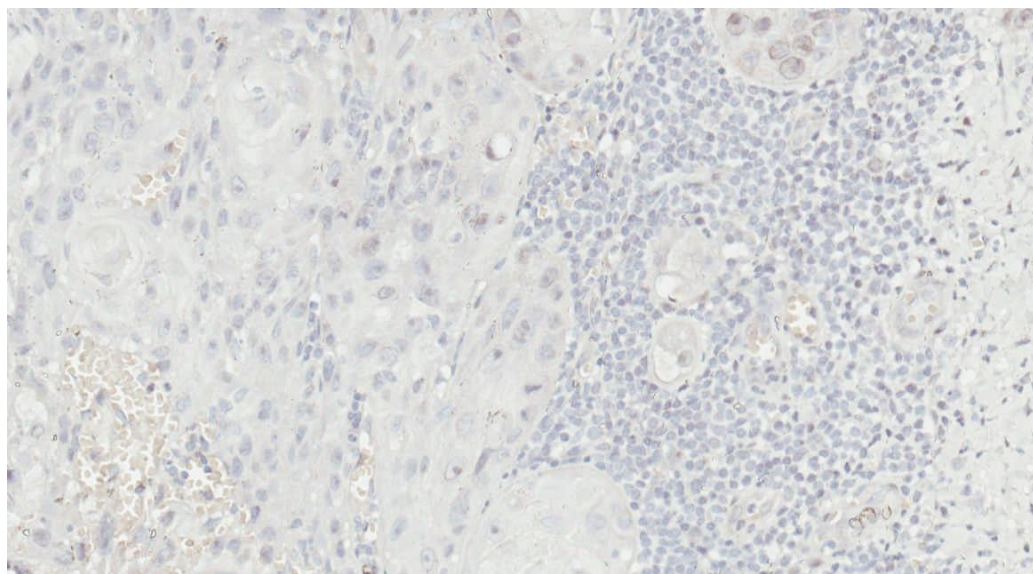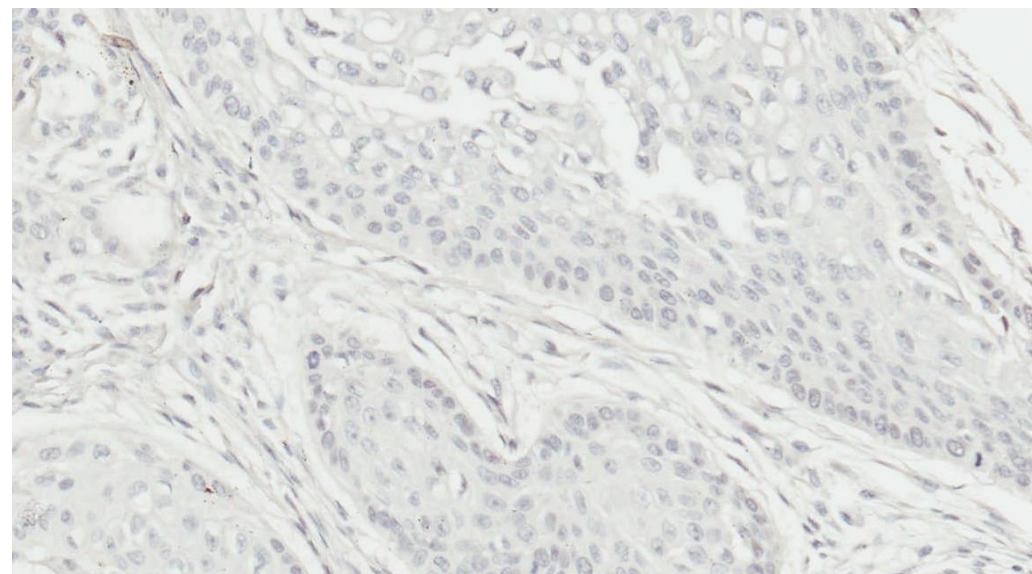

HNSC

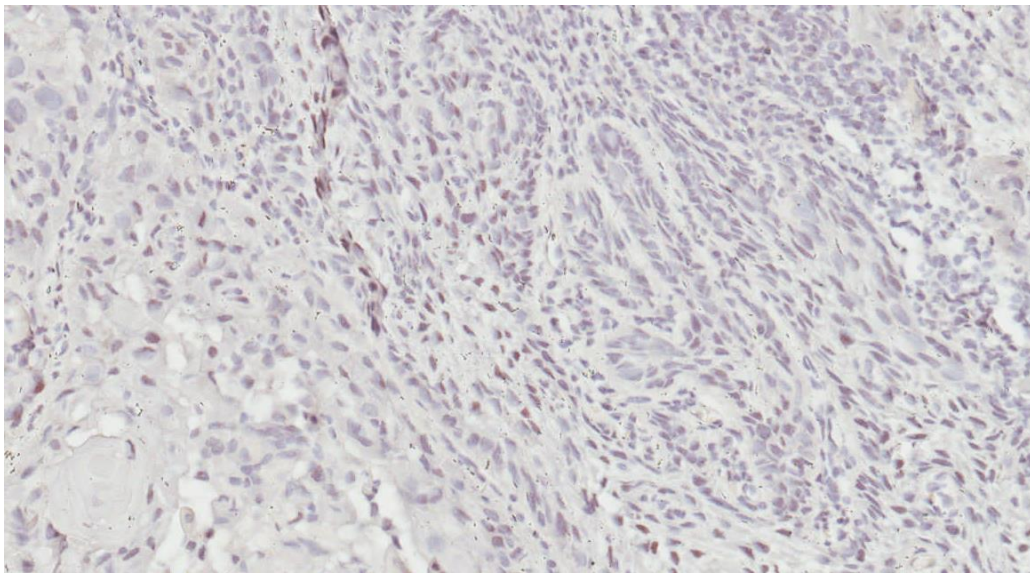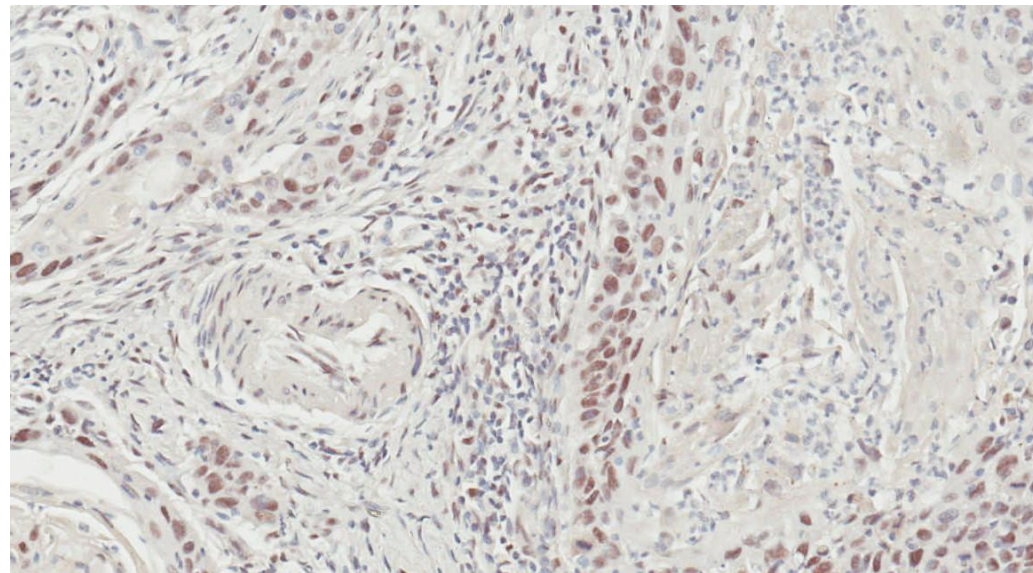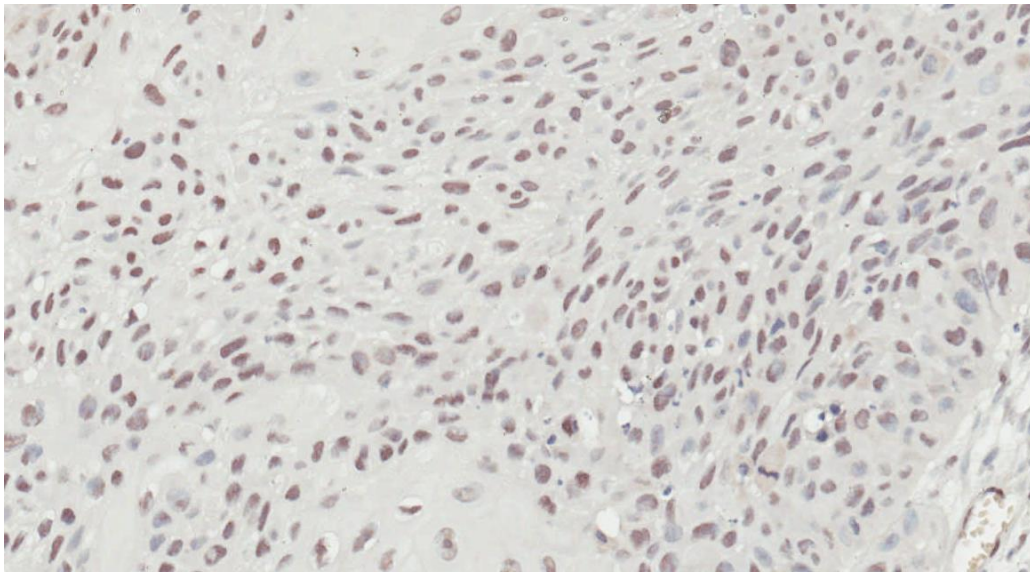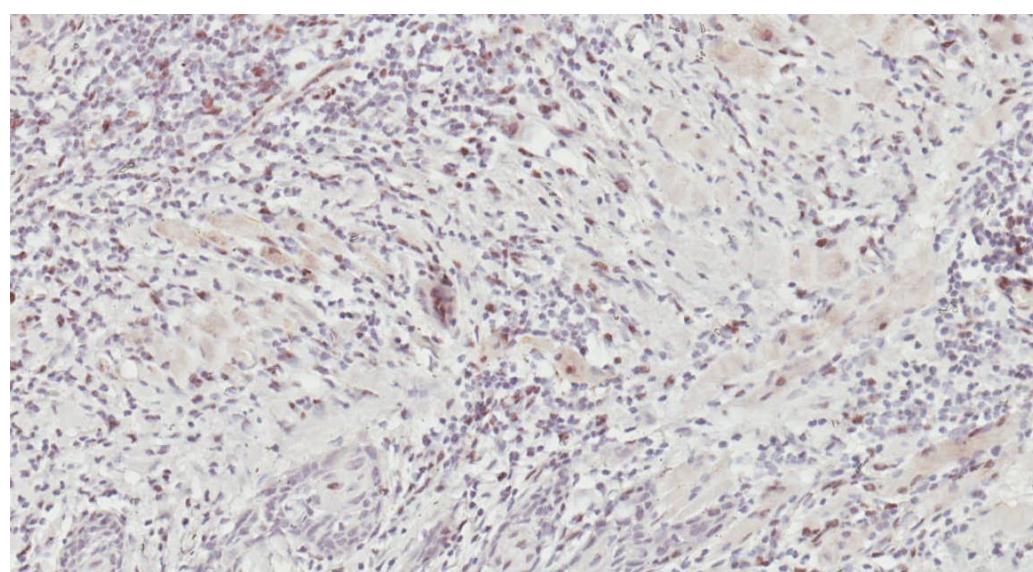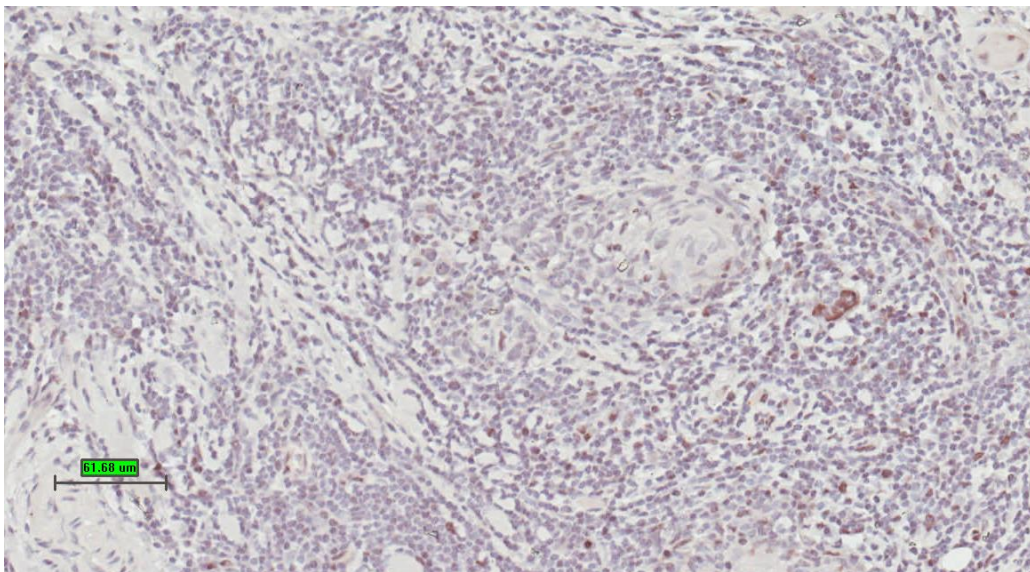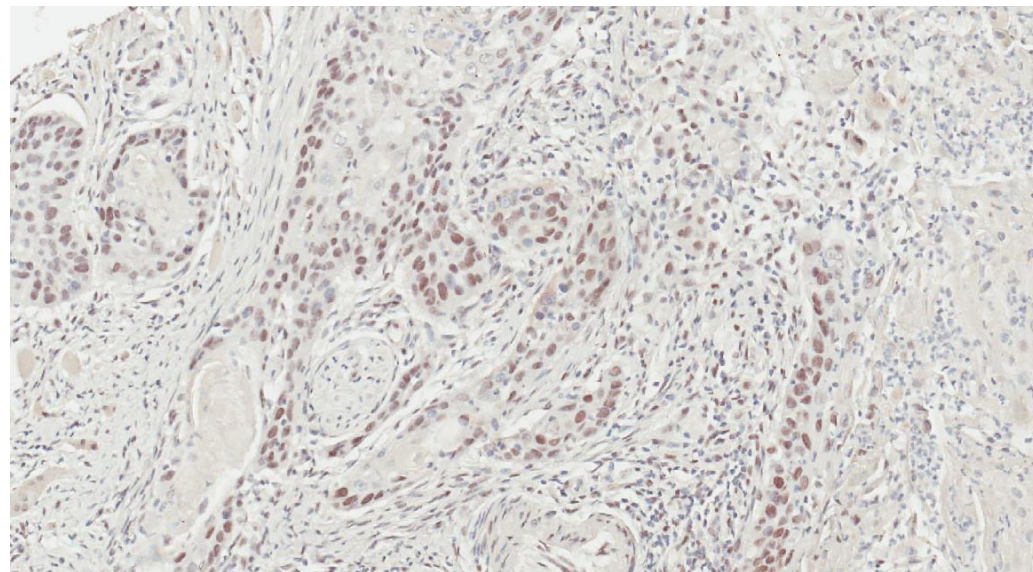

Supplement: Supplementary file 2 [file DataSheet_2.pdf]
